# Transformable H-bonds and conformation in compressed glucose†
†Electronic supplementary information (ESI) available: Detailed experimental data; detailed structural data. CCDC 1033994–1034008. For ESI and crystallographic data in CIF or other electronic format see DOI: 10.1039/c4sc03588g



**DOI:** 10.1039/c4sc03588g

**Published:** 2014-12-15

**Authors:** Ewa Patyk, Andrzej Katrusiak

**Affiliations:** a Department of Materials Chemistry , Faculty of Chemistry , Adam Mickiewicz University , Umultowska 89b , 61-614 Poznań , Poland . Email: katran@amu.edu.pl ; Fax: +48 618291555 ; Tel: +48 618291590

## Abstract

In response to external stimuli the α-d-glucose molecule extensively transforms its H-bonding pattern and conformation. High-pressure reverses the linear compressibility and changes the hierarchy of intermolecular interactions in crystalline α-d-glucose. At 5.40(2) GPa it undergoes an isostructural phase transition rearranging the OH···O hydrogen bonds, promoting the role of CH···O contacts and eliminating voids in the structure. Such monotonic and abrupt transformations may be characteristic of sugars and polysaccharides.

## Abstract 2

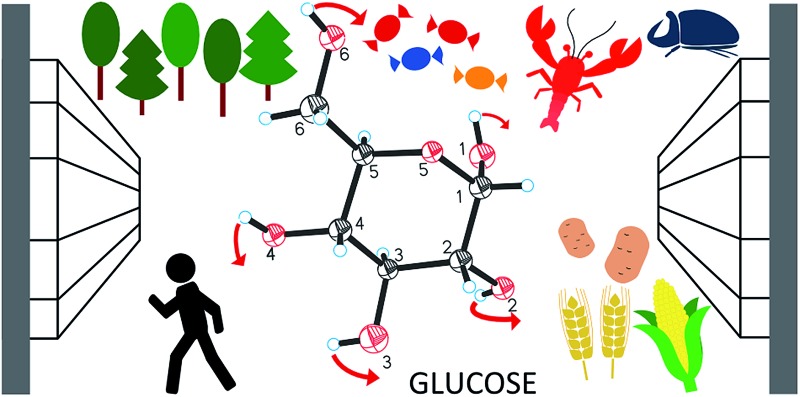
Upon pressurisation above 5.4 GPa, α-d-glucose transforms into a new polymorph, with an altered molecular conformation and with intermolecular hydrogen bonds reshuffled.

## Introduction

**Fig. 1:**
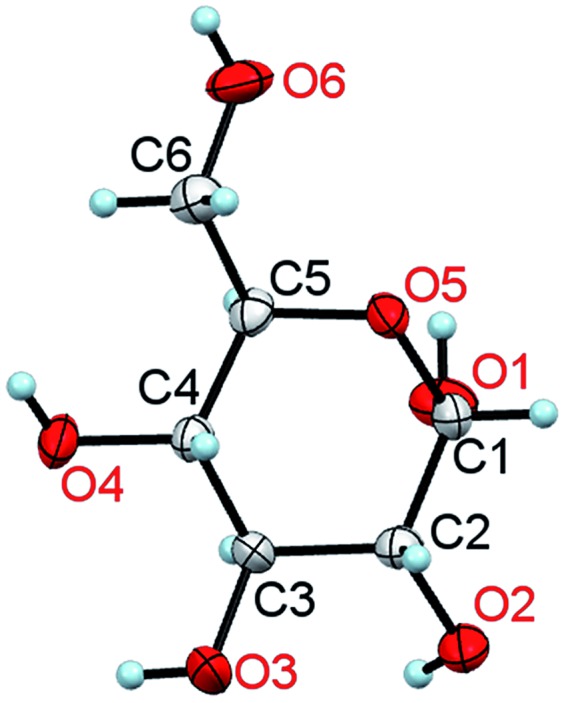
A molecule of α-d-glucose in the crystal at 295 K/0.1 MPa. The thermal ellipsoids are shown at the 50% probability level.^11^

Only water can compete with glucose in being the most significant substance in the living world. In its monomeric form, glucose (Fig. 1) is the main energy carrier in most living organisms and is their significant source of nutrition, being convertible into other fuels, such as lipids. Except for vitamins, amino acids and fatty acids, all vital metabolites can be obtained from glucose.^1^ Owing to their exceptional mechanical properties, glucose polymers have been chosen by Nature as the main building block of plants (cellulose) and for external skeletons of insects (chitin). It is the main raw material for the wood, food, construction, textile, paper, pharmaceutical and other industries.

However, despite playing such an important role, the mechanical properties of glucose itself have not been investigated and structural information about the glucose molecule, its transformations and interactions is still scarce. As early as 1913, soon after Laue's discovery, cellulose was studied by X-ray diffraction by Nishikawa and Ono.^2^ But it was several decades later that its structure built of OH···O bonded glucose polymers was postulated,^3^ and only more recent techniques allowed investigation of its structure and intermolecular interactions in detail.^4,5^ Most recently, conformational states of some isomers of d-glucopyranose α- and β-anomers in the gas phase were studied,^6,7^ but no solid-state transitions, neither of glucose nor of any other monosaccharide, have been reported so far. Such transitions could elucidate the interplay between the conformation of molecules and their interactions. Here we have employed high pressure as an external mechanical stimulus for transforming the intermolecular interactions of crystalline α-d-glucose. We show that pressure significantly modifies the molecular conformation and properties of the crystal and induces its new phase, unknown until now.

Like in H_2_O ices, the aggregation of glucose molecules in crystals is governed mainly by OH···O hydrogen bonds. It was first shown for the α-d-glucose monohydrate structure (monoclinic, space group *P*2_1_, *Z*′ = 2), determined in 1962 by Killean and Ferrier,^8^ in 1963 for β-d-glucose by Ferrier,^9^ and in 1965 for α-d-glucose by Brown and Levy^10^ (both orthorhombic, *P*2_1_2_1_2_1_, *Z*′ = 1). No solid-state phase transitions were reported for any of the glucose crystals so far. The presently reported phase transition at 5.40 GPa in α-d-glucose is the first one reported in monosaccharides and it provides detailed information on the structural and mechanical properties of these compounds. The new phase of α-d-glucose has been denoted as Phase II.

## Experimental

**Fig. 2:**
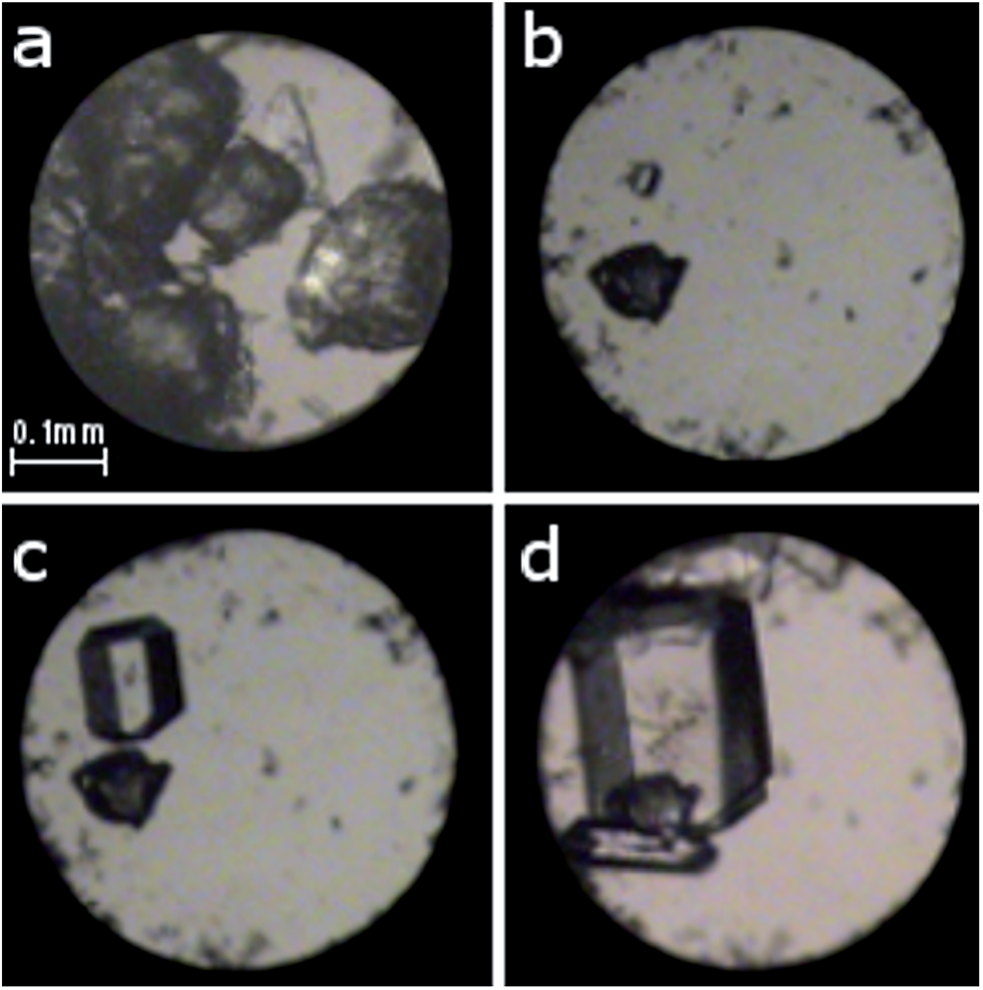
Isochoric growth of α-d-glucose single crystals from ethanol solution: (a) polycrystalline α-d-glucose after sealing the chamber; (b) a single seed at 383 K; (c) 343 K; (d) one larger and a few smaller crystals at 295 K/0.27(2) GPa. A ruby chip for pressure calibration lies close to the left edge of the chamber below the sample.

Two different approaches were applied in order to investigate the phase transitions and the formation of new polymorphs at high pressure. First, polycrystalline α-d-glucose (analytical grade from Sigma-Aldrich) and a small ruby chip were loaded into a modified Merrill-Bassett diamond-anvil cell;^12^ the tungsten gasket was 0.33 mm thick with a hole of 0.47 mm in diameter. The chamber was filled with ethanol and then compressed to 0.27(2) GPa. Several single crystals were obtained by gradually cooling the solution between 343 and 295 K (Fig. 2 and S1†). In the other approach, a single crystal of about 0.15–0.30 mm in size, obtained at ambient conditions, was mounted in the DAC along with the ruby chip and a cotton fibre, fixing the crystal position. Ethanol or methanol : ethanol : water (volume ratio 16 : 3 : 1) solutions saturated with d-glucose (to prevent dissolution of the sample) were used as hydrostatic media (Fig. S2†). The crystals were compressed to 0.88, 1.12, 1.36, 1.80, 2.60, 3.21, 4.00, 4.20, 5.15, 5.23, 5.33, 5.54, 5.80 and 6.20 GPa. The pressure inside the DAC was determined by the ruby-fluorescence method^13^ with a Photon Control Inc. spectrometer (to an accuracy of about 0.02 GPa). For each pressure point, the diffraction data were measured with a four-circle KUMA X-ray diffractometer with graphite monochromated Mo *Kα* radiation. Previously described procedures^14^ were applied for the crystal centring and data collection, UB-matrix determinations and data reductions were performed with the program CrysAlisPro^15^ and the program REDSHABS^16^ was used for the DAC absorption, gasket shadowing and sample absorption corrections. The programs SHELXS and SHELXL^17^ were used for solving and refining the structures.

The diffraction data showed that α-d-glucose is monotonically compressed in the orthorhombic space group *P*2_1_2_1_2_1_ to 5.40(2) GPa, above which it undergoes an isostructural phase transition. Experimental and crystallographic data of α-d-glucose as a function of pressure are listed in Table S1.†

In order to calculate the compressibility parameters *β*_*x*_ = –1/*x*∂*x*/∂*p*, where *x* is *V*, *a*, *b* or *c*, the differential part was analytically calculated based on a quadratic polynomial fitted to the measured unit-cell volume parameters for α-d-glucose Phase I and to a linear function for Phase II (Table S8†).

The α-d-glucose structure determined from neutron diffraction data^18^ was consistent with our X-ray diffraction study at 295 K/0.1 MPa, and it was used as the ambient-conditions reference. For the analyses and for comparing OH···O, CH···O and H···H contacts, the O–H and C–H bond lengths in the high-pressure structures have been normalized to the neutron-determined values according to Allan and Bruno.^19^

## Results and discussion

**Fig. 3:**
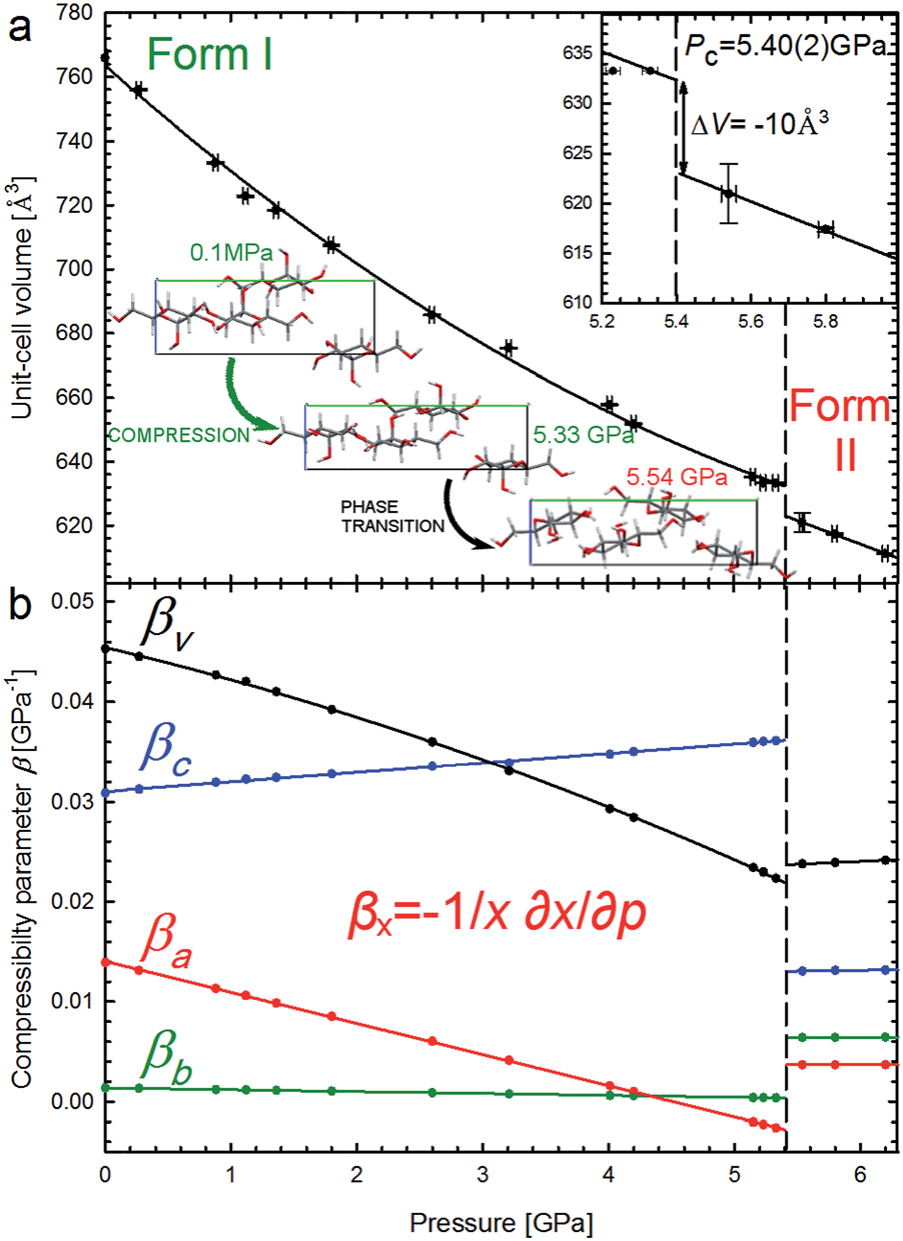
Unit-cell volume compression of the α-d-glucose Phases I and II (a), and linear compressibility coefficients *β* of the unit-cell parameters (b). The insets show an expanded view of the volume drop (Δ*V*) at the critical pressure (*P*_c_ – vertical dashed lines) and projections of the α-d-glucose unit cell along the [100] direction at 0.1 MPa, 5.33 GPa and 5.54 GPa, showing structural changes^11^ in the compressed Phase I, and after the phase transition in Phase II.

It is characteristic that the compression of α-d-glucose is strongly anisotropic and nonlinear to 5.4 GPa. α-d-Glucose is compressed monotonically at an average rate *β*_*V*_ = 34.64 × 10^–3^ GPa^–1^ up to *P*_c_ = 5.4 GPa (Fig. 3), upon which an anomalous drop of volume of 10 Å^3^ marks the isostructural phase transition.

This transition occurs when the crystal is compressed to about 80% of its ambient-pressure volume. The α-d-glucose forms below and above *P*_c_ have been denoted as Phases I and II, respectively. Their volume compression is similar, but the linear compression significantly changes (Fig. 3b). The softest is parameter *c* and its compressibility increases by 16.9% between 0.1 MPa and 5.4 GPa. The compression of parameter *b* is very small and practically constant within Phase I and that of parameter *a* is strongly nonlinear, gradually decreasing and eventually becoming negative at 4.6 GPa. It means that parameter *a* expands on approaching the high pressure limit of Phase I. It is characteristic of Phase II that its compression is much less anisotropic and that all its compressibility coefficients are almost constant to at least 6.2 GPa.

**Fig. 4:**
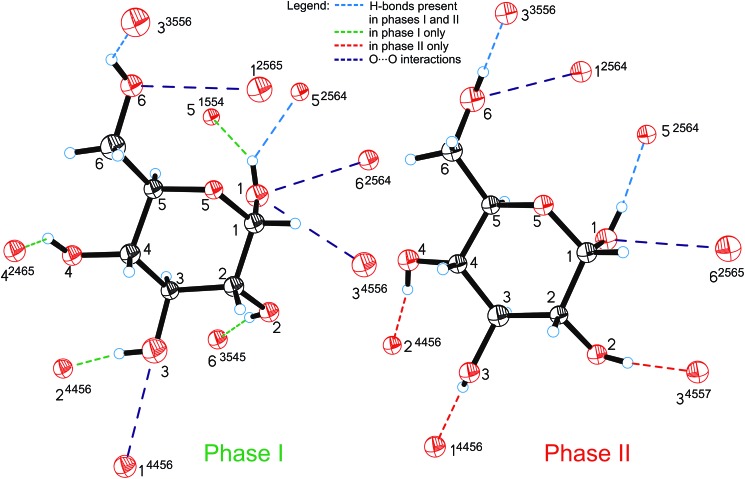
Hydrogen bonds OH···O (shown only from the H-donor side) and contacts O···O in α-d-glucose Phases I and II. The H-bonds which are unique for Phases I and II are shown in green and red, respectively; H-bonds present in both forms of glucose are marked in blue and O···O repulsing interactions in purple. A 4-digit ORTEP symmetry code^20^ shows the symmetry operation joining H-donors and acceptors and is explicitly described in Table S6.†

All the compression changes reflect the pressure-induced transformations of the molecular and crystal structure, and also systematic changes in the intermolecular interactions. The changes of the OH···O bonding pattern and in the molecular conformation between Phases I and II are illustrated in Fig. 4. The conformations changing most at the transition are those of the hydroxyl groups O2H, O3H and O4H, because their H-atoms change acceptors (the torsion angles are listed in Table S2†).

**Fig. 5:**
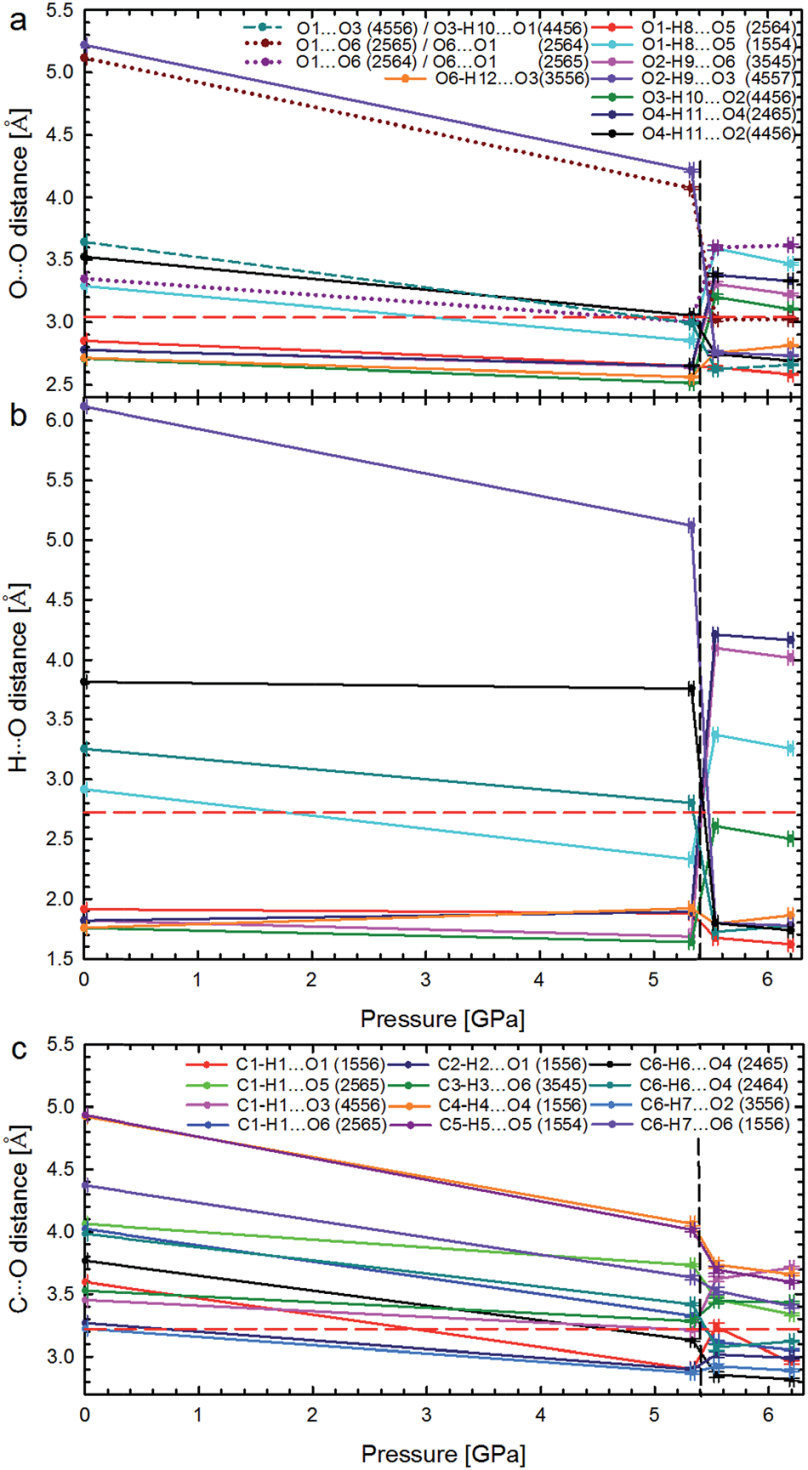
Pressure dependence of hydrogen bonds OH···O: (a) distances O···O and (b) H···O in Phases I and II; as well as (c) distances C···O in the shortest CH···O contacts. The phase transition is marked by vertical dashed lines, and the sums of the van der Waals radii for contacts O···O (3.04 Å), H···O (2.72 Å), and C···O (3.22 Å),^21^ by horizontal red dashed lines. In plot (a) the unfavourable contacts O···O are marked with dotted lines; the short-dashes mark the non-bonding O···O contact in α-d-glucose Phase I, becoming an H-bond above 5.40 GPa. Lines between points are for guiding the eye. Symmetry operations indicated by ORTEP codes^20^ are explicitly listed in Table S6.†

The intermolecular H-bonds O6H···O3^3556^ and O1H···O5^2564^ of Phase I are retained (the ORTEP code of symmetry transformations is explicitly explained in the ESI, in Table S6†), however in the bifurcated bond involving O1H, one contact, O1H···O5^1554^, is broken in Phase II. Hydroxyl groups O6H and O1H change their orientation in the sense preserving their short contacts. The torsion angle C5–C6–O6–H12, equal to *ca.* 180° in Phase I, changes its conformation, above 5.40 GPa, from *anti* to *anticlinal* (*ca.* –115°), adjusting itself to the position of H-acceptor. Similarly, torsion angle O5–C1–O1–H8 in Phase II adapts a +*gauche* conformation of *ca.* 60°, instead of the *synclinal* (*ca.* 30°) conformation present in Phase I. However, the positions of oxygen atoms O6 and O1 in the molecule alter only slightly at the phase transition, and their other interactions, including the unfavourable O···O contacts, are preserved above *P*_c_. There are two such contacts per molecule in Phase I. The molecular rearrangement leading to Phase II releases only one of them and the other, O1···O6^2564^/O6···O1^2565^, changes into O1···O6^2565^/O6···O1^2564^ (Fig. 5a). The pressure-induced rotation of group O3H transforms the O···O contact O1···O3^4556^/O3···O1^4456^ of Phase I into an H-bond O3H···O1^4456^ (Fig. 4, Table S3†).

**Fig. 6:**
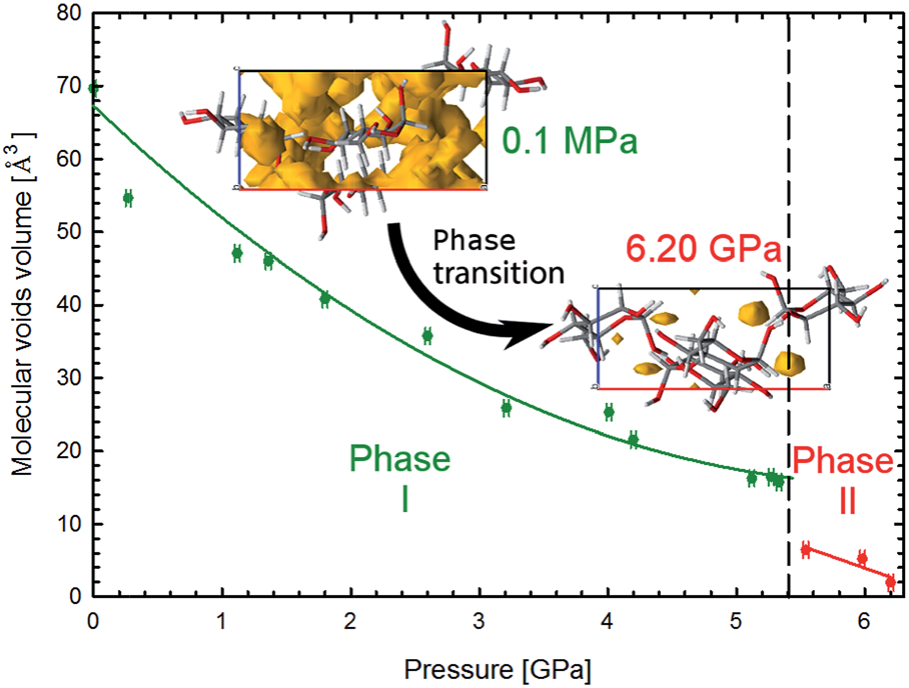
Pressure dependence of the molecular voids volume in α-d-glucose Phases I and II, marked in green and red, respectively. The insets show the molecular voids distribution along direction [010] at 0.1 MPa and 6.2 GPa. Voids marked in yellow have been calculated with probe radius and approximated grid spacing both of 0.4 Å.^11^

The strong rearrangement of OH···O and O···O contacts is also clearly visible in the relevant distances plotted *versus* pressure in Fig. 5. The shortest OH···O contacts are strongly reshuffled between Phases I and II, while the CH···O contacts are gradually compressed from ambient pressure, through the phase transition, and on in Phase II to at least 6.2 GPa. Between α-d-glucose Phases I and II the number of OH···O hydrogen bonds altogether decreases from 6 to 5 (Fig. 4, 5 and S4–S7 and Table S3†). Of the OH···O bonds of Phase I, two have been retained in Phase II, while four have been broken and in their stead three new ones have been formed. At the same time, eleven CH···O contacts, with H···O distances significantly shorter than the sum of the van der Waals radii,^21^ are formed after the phase transition (Fig. S8, Table S4†). This is a very strong increase, as only one such CH···O contact exists in the α-d-glucose Phase I structure at 0.1 MPa and eight are present at 5.33 GPa. The short CH···O contacts have been classified as such if their H···O distances are equal to or shorter than the sum of the van der Waals radii and the C–H···O angles are larger than 110°.^22^ Like in sucrose, the shortest O···O distance in the OH···O bonds of α-d-glucose Phase II is still longer than the shortest O···O distance of Phase I at 5.33 GPa (Fig. 5a and b). As for the CH···O contacts, their C···O distances (Fig. 5c) decrease with pressure, and the shortest C···O contact is in Phase II. Interestingly, the H···O distances of the shortest CH···O contacts in α-d-glucose become somewhat longer in Phase II, while for the shortest OH···O bonds of Phases I and II, the H···O distances are almost equal (Fig. S4, S8 and ESI†). The compression of α-d-glucose also affects the H···H contacts (Fig. S11†), which become shorter than those considered as dihydrogen and hydrogen–hydrogen bonding interactions.^23^ Their number increases considerably in the compressed Phase I, and then above the phase transition stays almost unchanged (Fig. S11, S14, Table S5†). However in both phases the H···H contacts become significantly shorter than the sum of the van der Waals radii. Thus the structural changes in the compressed crystal of α-d-glucose favour weak CH···O and H···H contacts (Fig. 5c, S8†), which can be interpreted as an increased role of van der Waals interactions, becoming competitive against OH···O hydrogen bonds. The OH···O bonds, *i.e.* the strongest interactions between the molecules, adjust to much weaker, although more numerous, CH···O and H···H contacts. In other words, the compression of intermolecular contacts and molecular rearrangements are governed by the transformable framework of OH···O bonds adjusting the molecular packing to the monotonically shrinking voids in α-d-glucose, and through the stepwise collapse between Phases I and II (Fig. 6, S15, S16†).

The crystal compression (Fig. 3) and the contraction of voids (Fig. 6, S16†) are very similar. Between 0.1 MPa and 5.4 GPa the unit cell of α-d-glucose is compressed by 133 Å^3^ and the largest voids decrease in volume by over 50 Å^3^ in this range. At the transition the volume drop of the unit cell and that of the voids are nearly equal, of 10 Å^3^. In α-d-glucose Phase II, up to 6.2 GPa the unit cell and its voids are compressed by almost 10 Å^3^ and 4.5 Å^3^, respectively. In this respect the transition of α-d-glucose can be classified as a structural collapse of a convertible OH···O bonds framework, leading to systematic changes in its pattern, adjusting to increasingly important CH···O interactions. These strong transformations of OH···O bonds, the gradual and consistent compression of CH···O contacts and the alterations of the OH···O and CH···O bonds hierarchy were also observed in compressed (+)-sucrose crystals.^24^

## Conclusions

The elements of pressure-induced structural transformations observed in detail in the α-d-glucose structure can also take place in glucose polymers, such as cellulose. Single polymeric strands of cellulose are interconnected similarly to glucose molecules. Therefore the transformations of OH···O bonds, as well as the stabilizing role of CH···O bonds, described for the α-d-glucose structure, can contribute to the exceptional elasticity of cellulose fibres. The structures of crystalline sugars are generally similar in molecular features and in the types of intermolecular interactions. The cohesion forces of all sugars are dominated by OH···O bonds, which are directional and aggregate the molecules into 3D frameworks. On the other hand, these OH···O bonds can be easily transformed owing to the rotations of hydroxyl groups, which allow reconstructions of the H-bonding networks. We have shown that external mechanical stimuli can destabilize the OH···O bonding by collapsing voids and favouring denser arrangements that are more dependent on other types of interactions, mainly CH···O bonds. It is apparent that the remarkable resemblance of interactions and aggregation of α-d-glucose and (+)-sucrose molecules, both governed by OH···O bonds, translate to their similar susceptibility to pressure. There are several striking analogies in the compression of these sugars. They both undergo isostructural phase transitions, α-d-glucose at 5.40 GPa and (+)-sucrose at 4.80 GPa, and the OH···O bonds are transformed and their role diminish, while CH···O bonds become more important. In both cases the transition takes place after compression of the unit-cell to about 80% of its ambient volume. These analogies suggest that sugars undergo similar transformations at high pressure due to their characteristic structural features, and similar transformations can be common in other OH···O bonded systems, abundant in Nature. Further high-pressure studies of sugars are still needed to better understand their elastic and mechanic properties.
