# Supplementary material for: Transformable H-bonds and conformation in compressed glucose
Source: Chem Sci. 2014 Dec 15;6(3):1991–5. doi: 10.1039/c4sc03588g (PMC5501096; doi:10.1039/c4sc03588g)
Supplement: Supplementary file 1 [file SC-006-C4SC03588G-s001.pdf]

Electronic Supplementary Material (ESI) for Chemical Science.
This journal is © The Royal Society of Chemistry 2014

Transformable H-bonds and conformation in compressed glucose

Ewa Patyk and Andrzej Katrusiak

Department of Materials Chemistry, Faculty of Chemistry, Adam Mickiewicz University

Umultowska 89b, 61-614 Poznań

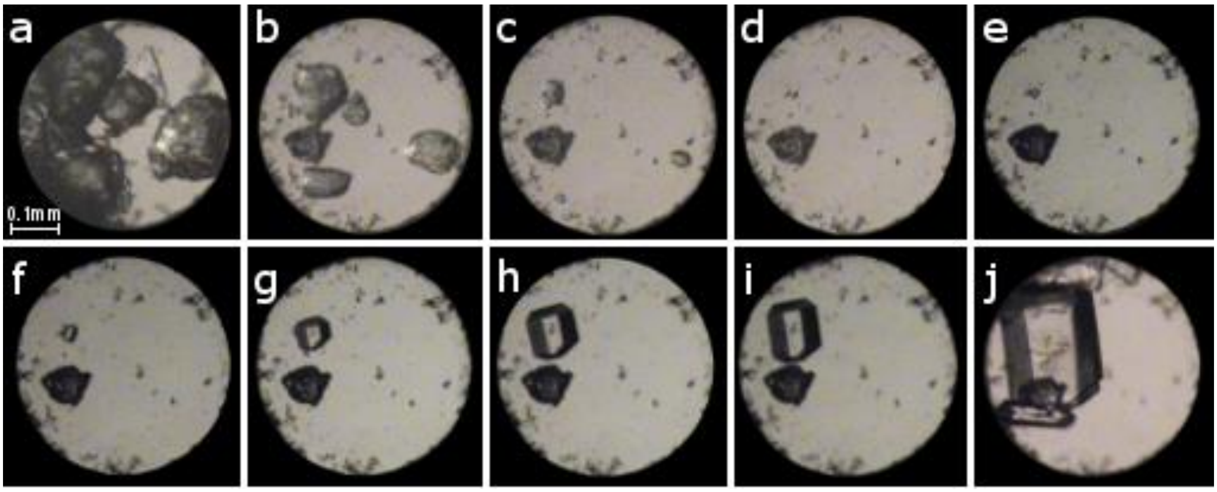

Fig. S1. Isochoric growth of α-D-glucose single crystals of ethanol solution: (a) polycrystalline mass at 295 K and 0.27(2) GPa; (b) few single crystals at 383 K; (c-e) one crystal seed at 383 K; (f-g, h-i) isochoric growth at 373 and 343 K respectively; one big single crystal and few small ones around the edges of gasket at 295 K and 0.27(2) GPa.

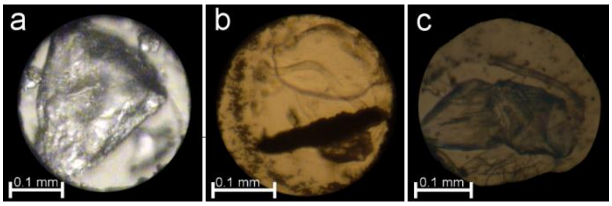

Fig. S2. Single crystals of α-D-glucose Phase I: at 295 K and (a) 0.88 GPa; Phase II (b) at 295 K and 5.27 GPa; (c) 5.54 GPa.

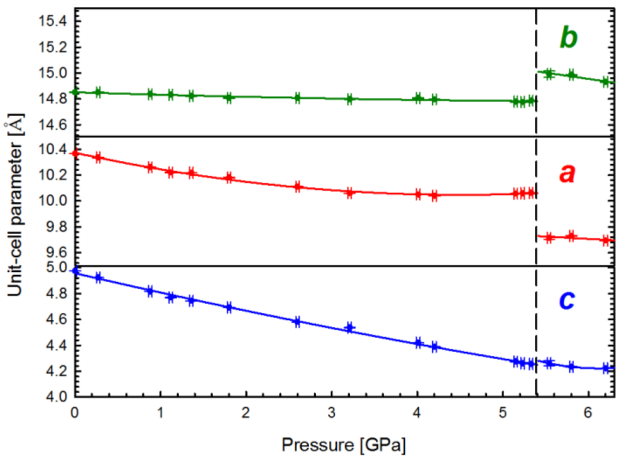

Fig. S3. The unit-cell parameters of α-D-glucose. The vertical dashed line marks the critical pressure at 5.40 GPa. Structural information at 0.1 MPa are cited after Brown and Levy.[1]

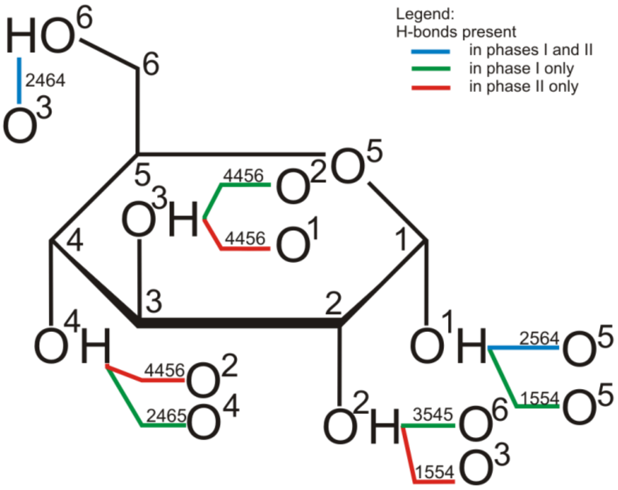

Fig. S4. H-bonding pattern in crystals of glucose, Phases I and II. H-bonds unique for Phase I and II are shown in green and red, respectively. H-bonds present in both Phases of glucose are marked in blue. Bond O3H···O2^{4456}, changing its polarity after phase transition to O2H···O3^{4457} is marked as a blue arrow. 4-Digit ORTEP symmetry code[2] showing symmetry operations for H-donors and acceptors are explicitly listed in Table S6.

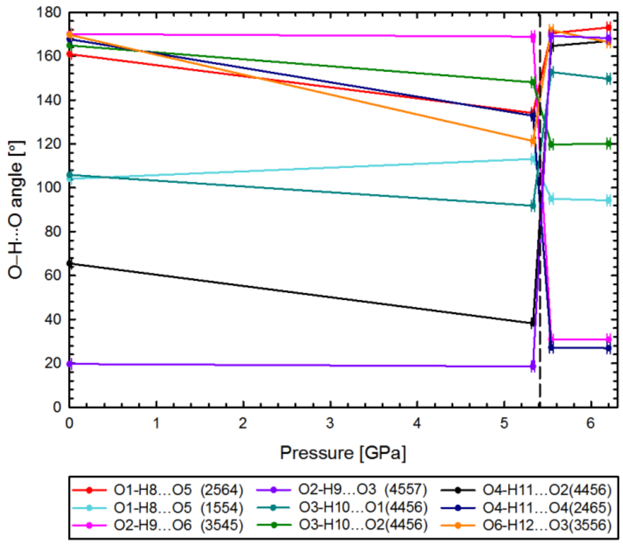

Fig. S5. Pressure dependence of O-H···O angles in O-H···O contacts. Critical pressure for phase transition is marked as vertical black dashed line. Lines joining points are shown to guide eye only. ORTEP code[2] used in the legend beneath the diagram is explicitly listed in Table S6.

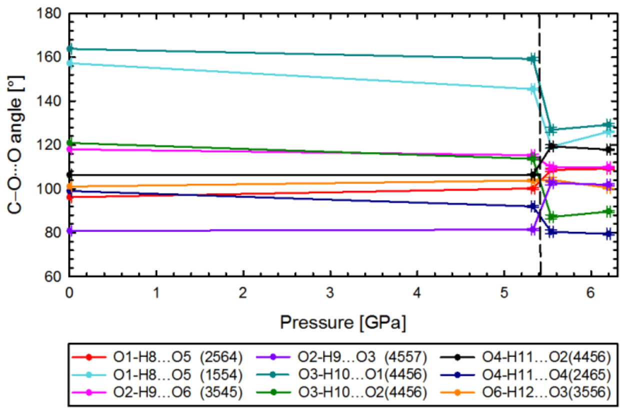

Fig. S6. Pressure dependence of C-O···O angles in O-H···O contacts. Critical pressure for phase transition is marked as vertical black dashed line. Lines joining points are shown to guide eye only. ORTEP code[2] used in the legend beneath the diagram is explicitly listed in Table S6.

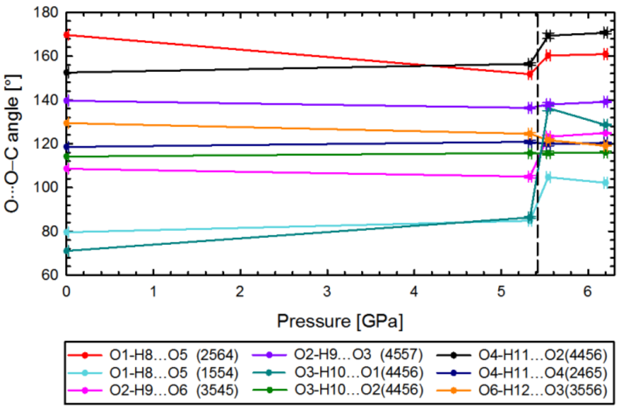

Fig. S7. Pressure dependence of O···O-C angles in O-H···O contacts. Critical pressure for phase transition is marked as vertical black dashed line. Lines joining points are shown to guide eye only. ORTEP code[2] used in the legend beneath the diagram is explicitly listed in Table S6.

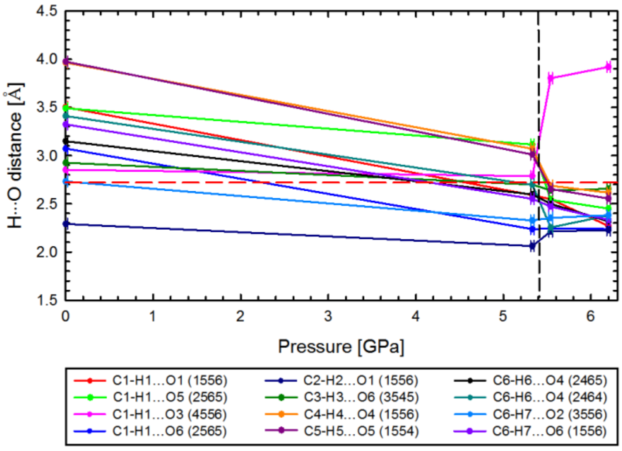

Fig. S8. Pressure dependence of H···O distance in C-H···O contacts. Critical pressure for phase transition is marked as vertical black dashed line and the sum of van der Waals radii of hydrogen and oxygen atoms, equal 2.72 Å,[3] as horizontal red dashed line. Lines joining points are shown to guide eye only. ORTEP code[2] used in the legend beneath the diagram is explicitly listed in Table S6.

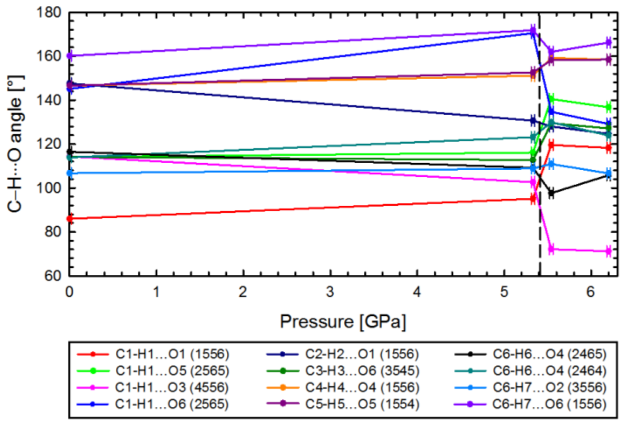

Fig. S9. Pressure dependence of C-H···O angles in C-H···O contacts. Critical pressure for phase transition is marked as vertical black dashed line. Lines joining points are shown to guide eye only. ORTEP code [2] used in the legend beneath the diagram is explicitly listed in Table S6.

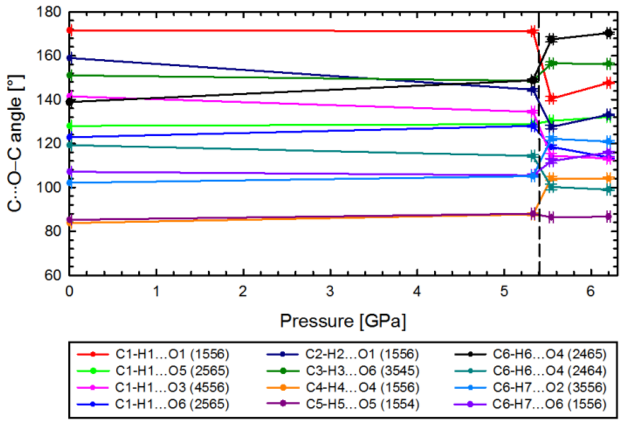

Fig. S10. Pressure dependence of C···O-C angles in C-H···O contacts. Critical pressure for phase transition is marked as vertical black dashed line. Lines joining points are shown to guide eye only. ORTEP code[2] used in the legend beneath the diagram is explicitly listed in Table S6.

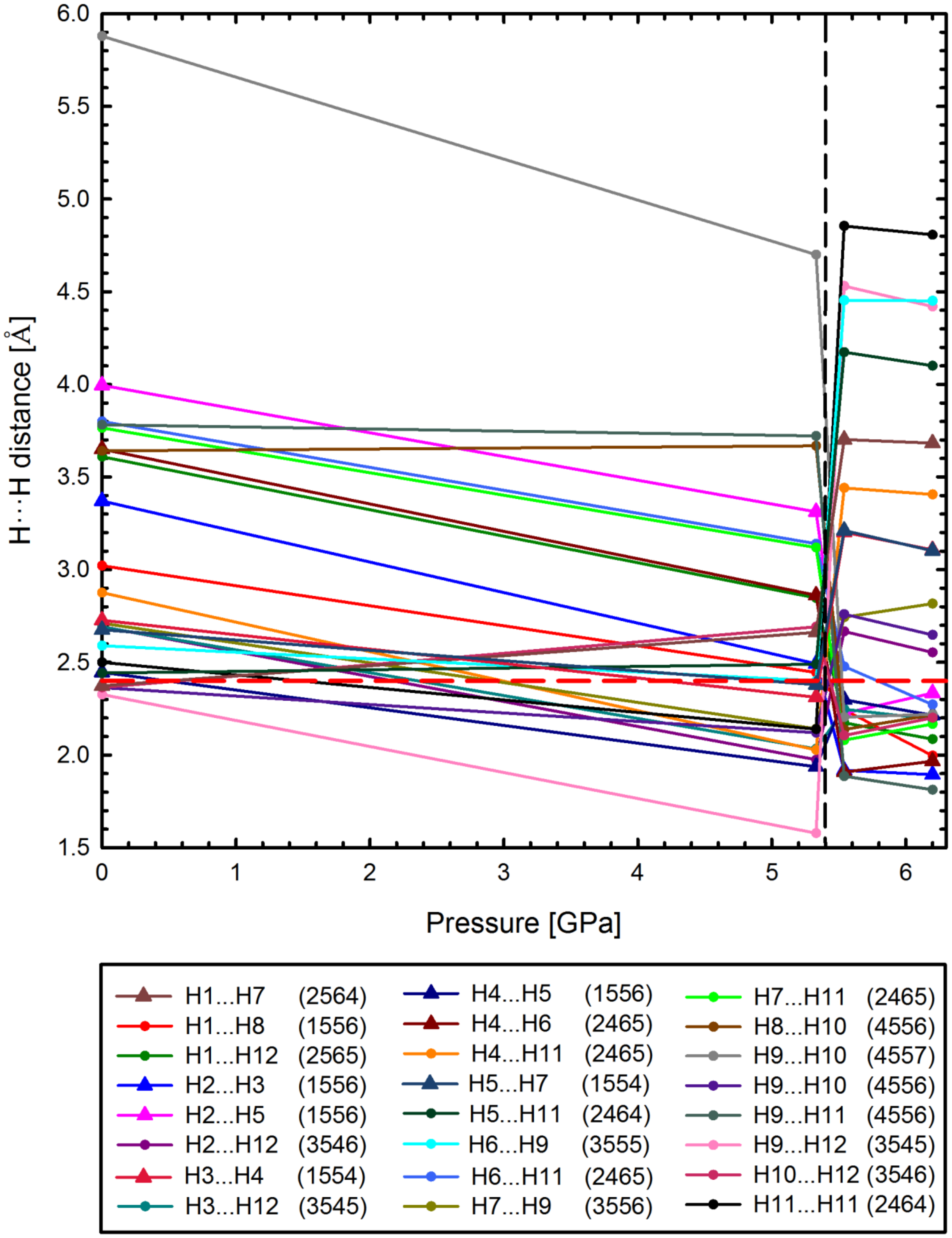

Fig. S11. Pressure dependence of H···H distance in H···H contacts. Critical pressure for phase transition is marked as vertical black dashed line and the sum of van der Waals radii of two hydrogen atoms, equal 2.40 Å,[3] as horizontal red dashed line. Lines joining points are shown to guide eye only. Lines joining points are shown to guide eye only. ORTEP code[2] used in the legend beneath the diagram is explicitly listed in Table S6.

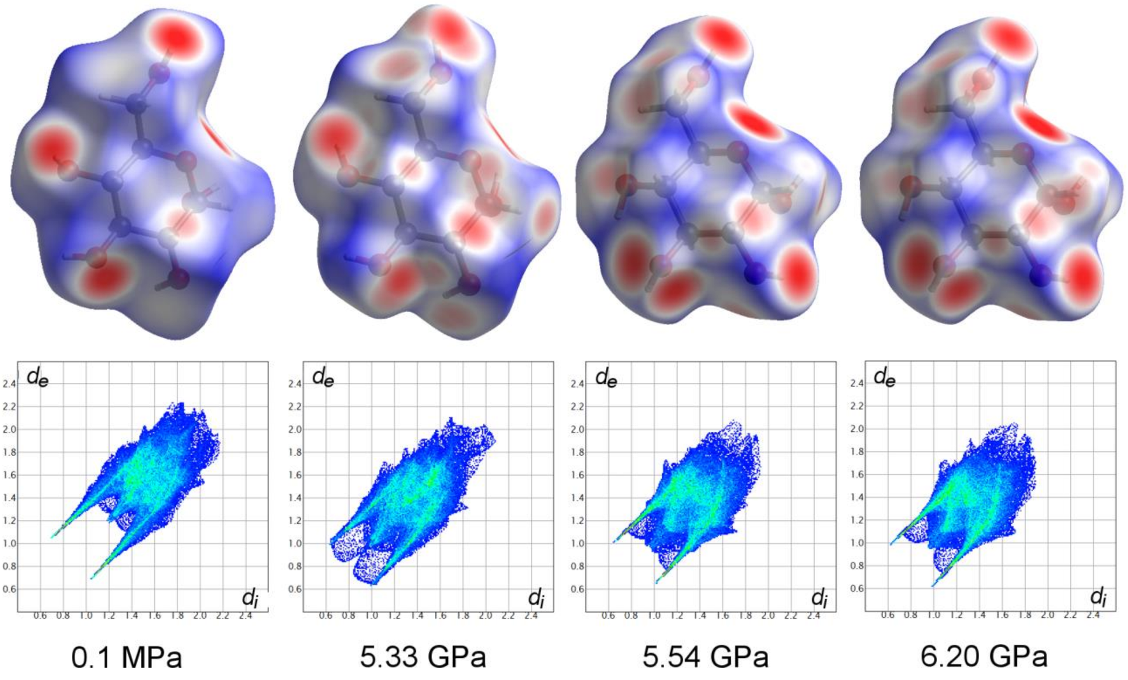

Fig. S12. 3D (top) and 2D fingerprint[4] (bottom) projections of Hirschfield surface[5] for glucose molecules of Phase I, at 0.1 MPa and 5.33 GPa, and Phase II at 5.54 and 6.20 GPa, mapped with d_{norm} *on* its natural range of –0.626 (red) and +1.379 (blue). For two-dimensional fingerprint plot blue corresponds to low frequency of a (d_i, d_e) pair and colour going through green to red shows increasing frequency.

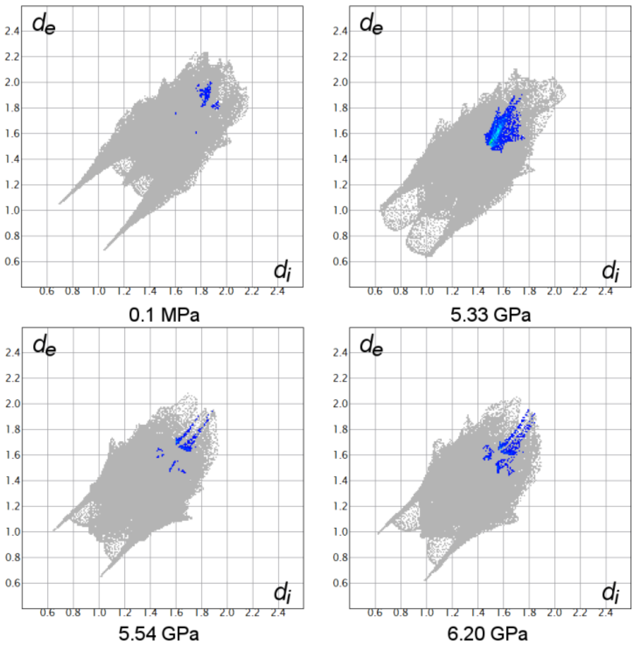

Fig. S13. Two-dimensional fingerprint plots[4] of sucrose Phase I, at 0.1 MPa and 5.33 GPa, and Phase II at 5.54 and 6.20 GPa, with (d_i, d_e) pairs marked for O···O contacts. Blue corresponds to low frequency of a (d_i, d_e) pair and color going to red shows increasing frequency.

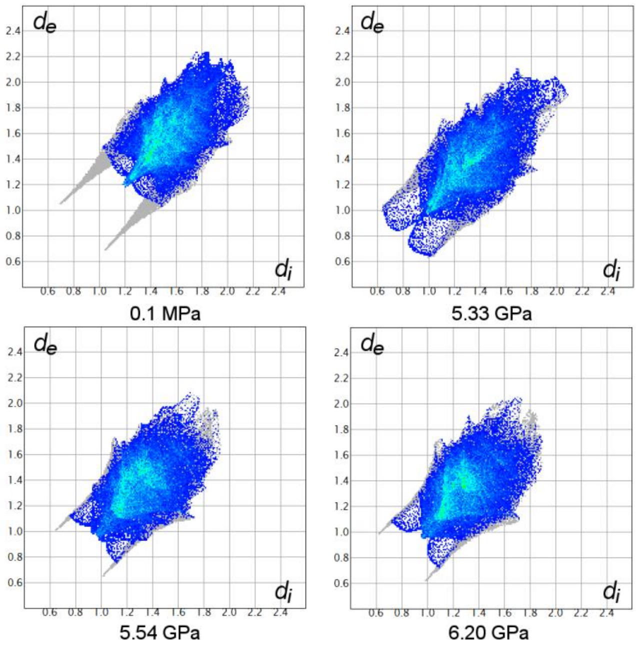

Fig. S14. Two-dimensional fingerprint plots[4] of sucrose Phase I, at 0.1 MPa and 5.33 GPa, and Phase II at 5.54 and 6.20 GPa, with (d_i, d_e) pairs marked for H···H contacts. Blue corresponds to low frequency of a (d_i, d_e) pair and color going to red shows increasing frequency.

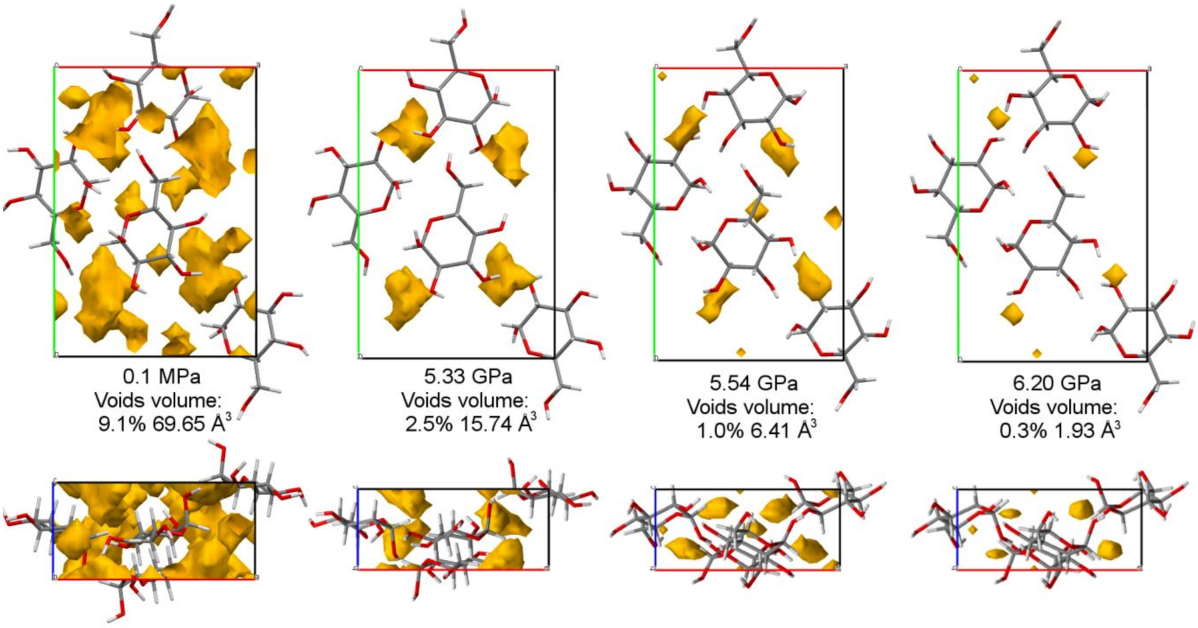

Fig. S15. Projection of glucose Phase I and II crystal structures along direction [001] (upper) and [010] (lower) with molecular voids (calculated with probe radius and approximated grid spacing of 0.4 Å) marked in yellow.[6] The exact values of molecular voids volume have been also included.

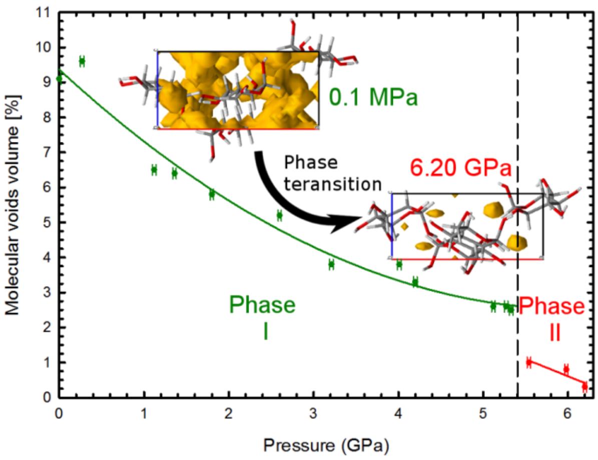

Fig. 16. Pressure dependence of molecular voids volume in in α-D-glucose Phases I and II, marked in green and red, respectively. Projection of molecular voids distribution along direction [010] at 0.1 MPa and 6.2 GPa has been also included. Voids marked in yellow have been calculated with probe radius and approximated grid spacing of 0.4 Å.[6]

Table S1. Selected experimental data for α-D-glucose Phases I and II.

$C_{12}H_{22}O_{11}$	Phase I	Phase I	Phase I	Phase I	Phase I	Phase I	Phase I	Phase I
Pressure (GPa)	0.27(2)	0.88(2)	1.12(2)	1.36(2)	1.80(2)	2.60(2)	3.21(2)	4.00(2)
Temperature (K)	295(2)	295(2)	295(2)	295(2)	295(2)	295(2)	295(2)	295(2)
Formula weight	180.16	180.16	180.16	180.16	180.16	180.16	180.16	180.16
Crystal colour	Colourless	Colourless	Colourless	Colourless	Colourless	Colourless	Colourless	Colourless
Crystal size (mm)	0.31x0.29x0.20	0.33x0.27x0.20	0.33x0.25x0.20	0.33x0.25x0.20	0.33x0.25x0.20	0.20x0.19x0.16	0.20x0.19x0.16	0.20x0.19x0.16
Crystal system	Orthorhombic	Orthorhombic	Orthorhombic	Orthorhombic	Orthorhombic	Orthorhombic	Orthorhombic	Orthorhombic
Space group	$P2_12_12_1$	$P2_12_12_1$	$P2_12_12_1$	$P2_12_12_1$	$P2_12_12_1$	$P2_12_12_1$	$P2_12_12_1$	$P2_12_12_1$
Unit cell dimensions (Å; °)								
a =	10.336(2)	10.260(2)	10.220(2)	10.218(2)	10.181(2)	10.110(2)	10.060(2)	10.050(2)
b =	14.852(3)	14.837(3)	14.832(3)	14.823(3)	14.808(3)	14.808(3)	14.798(3)	14.807(3)
c =	4.9240(10)	4.8169(10)	4.7689(10)	4.7439(9)	4.6930(9)	4.5801(9)	4.5370(9)	4.4206(9)
Volume (Å^3)	755.9(3)	733.3(3)	722.9(3)	718.5(2)	707.5(2)	685.7(2)	675.4(2)	657.8(2)
Z	4	4	4	4	4	4	4	4
D_x (g cm^{-3})	1.583	1.632	1.655	1.665	1.691	1.745	1.772	1.819
Wavelength MoKα, λ (Å)	0.71073	0.71073	0.71073	0.71073	0.71073	0.71073	0.71073	0.71073
Absorption coefficient (mm^{-1})	0.144	0.148	0.150	0.151	0.153	0.158	0.161	0.165
F(000) (e)	384	384	384	384	384	384	384	384
2θ max (°)	53.56	53.60	53.88	54.16	55.50	51.48	51.12	51.66
Min./Max. indices h	-6/6	-12/11	-12/12	-11/12	-13/12	-2/2	-2/2	-2/2
k	-16/16	-13/13	-12/13	-14/13	-14/14	-17/17	-17/17	-18/18
l	-6/6	-4/4	-4/4	-4/4	-4/4	-5/5	-5/5	-5/5
Reflections collected/unique	2263/404	1752/484	1930/549	2098/526	1973/494	1626/279	1566/242	1532/204
R_{int}	0.0884	0.1006	0.0677	0.0930	0.0926	0.1322	0.1280	0.1358
Observed reflections (I>4σ(I))	340	394	426	443	407	191	159	148
Data/parameters	404/49	484/44	549/ 49	526/49	494/49	279/49	242/44	204/44
Goodness of fit on F^2	1.016	1.006	1.023	1.007	1.000	1.012	1.009	1.028
Final R_1 indices (I>4σ(I))	0.0642	0.0477	0.0626	0.0616	0.0612	0.0393	0.0541	0.0543
R_1/wR_2 indices (all data)	0.0718/0.1331	0.0571/0.0957	0.0728/0.1538	0.0694/0.1346	0.0689/0.1428	0.0595/0.0720	0.0758/0.1026	0.0761/0.1075
$\Delta\sigma_{max}$, $\Delta\sigma_{min}$ (eÅ^{-3})	0.22, -0.18	0.169,-0.146	0.280,-0.228	0.256,-0.240	0.268,-0.209	0.104,-0.120	0.126,-0.126	0.115,-0.093
Weighting scheme[a]: x; y	0.0853; 0	0.0421; 0	0.0999;0	0.0843; 0	0.0894; 0	0.0163; 0	0.0413; 0	0.0482; 0
Extinction coefficient	–	–	–	–	–	–	–	–
Absorption corrections	DAC, gasket and sample crystal	DAC, gasket and sample crystal	DAC, gasket and sample crystal	DAC, gasket and sample crystal	DAC, gasket and sample crystal	DAC, gasket and sample crystal	DAC, gasket and sample crystal	DAC, gasket and sample crystal
DAC transmission min/max	0.92 / 1.00	0.91/ 1.00	0.92 / 1.00	0.91 / 1.00	0.92 / 1.00	0.92 / 1.00	0.92 / 1.00	0.92 / 1.00
Gasket shadowing min/max	0.56 / 0.94	0.50 / 0.94	0.54 / 0.95	0.52 / 0.94	0.51 / 0.95	0.43 / 0.93	0.46 / 0.92	0.45 / 0.92
Sample transmission min/max	0.95 / 0.96	0.95 / 0.96	0.95 / 0.96	0.95 / 0.96	0.95 / 0.96	0.95 / 0.95	0.95 / 0.95	0.94 / 0.95

[a] $w = 1/(\sigma^2(F_o^2)+x^2P^2+yP)$, where $P=(\mathrm{Max}(F_o^2, 0)+2F_c^2)/3$

Table S1 (Continuation). Selected experimental data for α-D-glucose Phases I and II.

$C_{12}H_{22}O_{11}$	Phase I	Phase I	Phase I	Phase I	Phase II	Phase II	Phase II
Pressure (GPa)	4.20(2)	5.15(2)	5.23(2)	5.33(2)	5.54(2)	5.80(2)	6.20(2)
Temperature (K)	295(2)	295(2)	295(2)	295(2)	295(2)	295(2)	295(2)
Formula weight	180.16	180.16	180.16	180.16	180.16	180.16	180.16
Crystal colour	Colourless	Colourless	Colourless	Colourless	Colourless	Colourless	Colourless
Crystal size (mm)	0.20x0.19x0.16	0.20x0.15x0.10	0.20x0.15x0.10	0.20x0.15x0.10	0.10x0.10x0.08	0.15x0.10x0.10	0.15x0.10x0.10
Crystal system	Orthorhombic	Orthorhombic	Orthorhombic	Orthorhombic	Orthorhombic	Orthorhombic	Orthorhombic
Space group	$P2_12_12_1$	$P2_12_12_1$	$P2_12_12_1$	$P2_12_12_1$	$P2_12_12_1$	$P2_12_12_1$	$P2_12_12_1$
Unit cell dimensions (Å; °)							
a =	10.040(2)	10.056(2)	10.059(2)	10.063(2)	9.714(11)	9.7300(19)	9.6940(19)
b =	14.796(3	14.779(3)	14.776(3)	14.787(3)	14.99(2)	14.987(3)	14.931(3)
c =	4.3879(9)	4.2750(9)	4.2610(9)	4.2560(9)	4.262(19)	4.2340(8)	4.2234(8)
Volume (Å^3)	651.8(2)	635.3(2)	633.3(2)	633.3(2)	621(3)	617.4(2)	611.3(2)
Z	4	4	4	4	4	4	4
D_x (g cm^{-3})	1.836	1.883	1.889	1.890	1.928	1.938	1.958
Wavelength MoKα, λ (Å)	0.71073	0.71073	0.71073	0.71073	0.71073	0.71073	0.71073
Absorption coefficient (mm^{-1})	0.167	0.171	0.171	0.171	0.175	0.176	0.178
F(000) (e)	384	384	384	384	384	384	384
2θ max (°)	52.02	51.84	52.64	51.80	49.56	52.04	53.50
Min./Max. indices h	-1/1	-10/10	-10/10	-10/10	-11/11	-11/11	-12/12
k	-15/16	-17/16	-16/16	-17/16	-15/15	-15/15	-15/15
l	-5/5	-3/3	-3/3	-3/3	-2/2	-2/2	-2/2
Reflections collected/unique	1173/153	1805/471	1702/439	1592/441	1275/323	1737/371	1924/396
R_{int}	0.1119	0.1111	0.1130	0.1229	0.2050	0.1140	0.1008
Observed reflections (I>4σ(I))	116	297	297	270	197	258	305
Data/parameters	153/44	471/49	439/49	441/49	323/39	371/49	396/49
Goodness of fit on F^2	1.036	1.004	1.004	1.012	1.007	1.013	1.002
Final R_1 indices (I>4σ(I))	0.0389	0.0576	0.0614	0.0561	0.0674	0.0538	0.0523
R_1/wR_2 indices (all data)	0.0548/0.0677	0.0843/0.1107	0.0826/0.1314	0.0866/0.1040	0.0887/0.1489	0.0709/0.1032	0.0615/0.1023
$\Delta\sigma_{max}$, $\Delta\sigma_{min}$ (eÅ^{-3})	0.069,-0.069	0.217,-0.209	0.221,-0.283	0.191,-0.220	0.204,-0.204	0.187,-0.225	0.176,-0.173
Weighting scheme[a]: x; y	0.0222; 0	0.0390; 0	0.0570; 0	0.0300; 0	0.0484; 0	0.0410; 0	0.0530; 0
Extinction coefficient	–	–	–	–	–	–	–
Absorption corrections	DAC, gasket and sample crystal	DAC, gasket and sample crystal	DAC, gasket and sample crystal	DAC, gasket and sample crystal	DAC, gasket and sample crystal	DAC, gasket and sample crystal	DAC, gasket and sample crystal
DAC transmission min/max	0.92 / 1.00	0.92 / 1.00	0.92 / 1.00	0.92 / 1.00	0.92 / 1.00	0.92 / 1.00	0.92 / 1.00
Gasket shadowing min/max	0.45 / 0.91	0.45 / 0.92	0.46/ 0.92	0.44 / 0.92	0.38 / 0.88	0.39 / 0.89	0.37 / 0.92
Sample transmission min/max	0.94 / 0.95	0.94 / 0.95	0.94 / 0.95	0.94 / 0.95	0.94 / 0.95	0.94 / 0.95	0.94 / 0.95

[a] $w = 1/(\sigma_2(F_{o2})+x_2P_2+yP)$, where $P=(\mathrm{Max}(F_{o2}, 0)+2F_{c2})/3$

Table S2. Torsion angles in α-D-glucose Phases I and II.

Torsion angle	0.27(2)	0.88(2)	1.12(2)	1.36(2)	1.80(2)	2.60(2)	3.21(2)	4.00(2)
O1-C1-C2-O2	61.00(100)	57.32(56)	59.03(60)	56.61(68)	57.77(74)	60.50(160)	55.28(244)	58.86(210)
O5-C1-C2-O2	176.41(75)	178.81(43)	179.51(45)	178.15(51)	178.60(55)	-178.03(109)	179.43(173)	-179.31(139)
O1-C1-C2-C3	-65.67(111)	-70.17(68)	-70.28(67)	-72.00(76)	-71.24(83)	-76.98(162)	-81.07(244)	-76.64(219)
O5-C1-C2-C3	49.74(79)	51.31(67)	50.20(69)	49.54(85)	49.59(93)	44.50(110)	43.09(168)	45.19(158)
O2-C2-C3-O3	63.80(86)	62.36(69)	61.78(76)	61.31(84)	62.87(97)	61.43(123)	58.88(135)	62.50(151)
C1-C2-C3-O3	-170.82(54)	-171.27(54)	-169.87(58)	-170.42(71)	-169.83(79)	-168.23(42)	-167.34(63)	-167.51(66)
O2-C2-C3-C4	-178.63(55)	-177.29(52)	-178.11(50)	-177.42(59)	-176.25(68)	-176.43(72)	-174.44(83)	-171.52(83)
C1-C2-C3-C4	-53.25(93)	-50.93(65)	-49.75(69)	-49.15(81)	-48.94(92)	-46.09(135)	-40.66(149)	-41.53(150)
O3-C3-C4-O4	-68.33(91)	-66.52(62)	-65.25(61)	-66.43(73)	-65.78(87)	-59.33(162)	-66.24(259)	-62.81(219)
C2-C3-C4-O4	175.02(73)	176.20(49)	176.55(53)	174.05(58)	175.02(67)	178.36(165)	173.93(167)	174.86(130)
O3-C3-C4-C5	172.59(76)	172.99(39)	173.98(42)	174.25(47)	174.47(53)	176.80(187)	177.74(352)	178.95(213)
C2-C3-C4-C5	55.94(95)	55.71(66)	55.78(67)	54.72(75)	55.27(87)	54.49(224)	57.90(364)	56.63(249)
O4-C4-C5-O5	-177.77(62)	-177.27(51)	179.92(54)	-177.75(60)	-179.20(70)	-179.96(77)	-178.34(107)	176.63(098)
C3-C4-C5-O5	-58.45(82)	-58.76(59)	-61.37(59)	-59.94(65)	-61.25(73)	-54.61(161)	-62.73(349)	-65.10(229)
O4-C4-C5-C6	62.44(87)	64.39(66)	63.93(63)	63.42(73)	63.88(81)	58.93(125)	63.93(258)	66.85(230)
C3-C4-C5-C6	-178.23(84)	-177.1(49)	-177.36(49)	-178.77(57)	-178.18(64)	-175.72(155)	179.54(339)	-174.88(262)
O5-C5-C6-O6	70.27(90)	70.48(58)	71.05(63)	70.27(71)	71.64(74)	65.81(118)	62.43(181)	75.86(235)
C4-C5-C6-O6	-173.19(72)	-172.37(41)	-172.61(44)	-171.81(53)	-172.60(58)	-177.31(191)	175.67(348)	-175.77(251)
C6-C5-O5-C1	-174.63(56)	-179.73(48)	-178.16(46)	-176.77(52)	-177.54(53)	-179.19(72)	-177.15(108)	-173.82(118)
C4-C5-O5-C1	64.83(84)	60.38(62)	63.76(63)	62.03(73)	63.93(79)	60.60(98)	58.84(136)	64.18(111)
O1-C1-O5-C5	52.55(123)	63.20(60)	60.51(62)	62.54(66)	60.44(73)	64.75(222)	69.74(318)	58.89(242)
C2-C1-O5-C5	-60.23(92)	-58.57(62)	-58.47(69)	-57.94(80)	-58.73(87)	-54.00(131)	-53.14(176)	-59.33(129)
O5-C1-O1-H8	40.63	30.61	32.61	31.07	31.79	29.12	28.06	34.05
C1-C2-O2-H9	-105.59	-105.19	-106.60	-106.59	-107.75	-114.77	-112.41	-114.35
C2-C3-O3-H10	178.64	179.42	179.22	179.57	179.21	-179.60	-179.79	177.73
C3-C4-O4-H11	174.17	173.41	173.49	173.92	173.45	174.34	170.98	170.30
C5-C6-O6-H12	177.92	175.43	177.48	178.44	178.53	-179.19	-179.83	176.63

Table S2 (Continuation). Torsion angles in α-D-glucose Phases I and II.

Torsion angle	4.20(2)	5.15(2)	5.23(2)	5.33(2)	5.54(2)	5.80(2)	6.20(2)
O1-C1-C2-O2	63.64(188)	51.96(93)	51.12(108)	50.85(97)	37.55(177)	48.39(89)	48.92(74)
O5-C1-C2-O2	-176.56(143)	174.38(77)	173.17(92)	173.97(91)	175.06(153)	170.59(89)	168.19(78)
O1-C1-C2-C3	-79.87(206)	-72.77(88)	-71.96(106)	-74.66(96)	-79.49(165)	-75.10(92)	-76.12(78)
O5-C1-C2-C3	39.93(179)	49.64(119)	50.08(148)	48.46(133)	58.02(177)	47.11(134)	43.15(118)
O2-C2-C3-O3	59.76(187)	67.76(090)	69.68(104)	67.23(104)	71.00(171)	70.34(104)	69.80(92)
C1-C2-C3-O3	-166.59(66)	-169.19(77)	-168.16(98)	-168.27(88)	-172.34(117)	-166.21(79)	-164.61(65)
O2-C2-C3-C4	-172.14(113)	-170.77(74)	-171.53(89)	-170.19(79)	-167.04(143)	-167.32(71)	-167.28(59)
C1-C2-C3-C4	-38.50(157)	-47.72(117)	-49.37(144)	-45.69(132)	-50.38(197)	-43.88(138)	-41.68(117)
O3-C3-C4-O4	-58.36(194)	-66.05(101)	-68.26(125)	-65.34(113)	-67.55(217)	-66.20(120)	-67.28(94)
C2-C3-C4-O4	174.51(132)	174.76(78)	174.92(90)	173.14(080)	174.28(153)	172.58(85)	170.79(69)
O3-C3-C4-C5	175.77(174)	172.03(80)	170.52(96)	173.54(85)	167.59(141)	170.65(78)	171.90(66)
C2-C3-C4-C5	48.64(199)	52.83(103)	53.71(124)	52.02(117)	49.41(215)	49.43(127)	49.97(108)
O4-C4-C5-O5	177.89(113)	178.06(80)	179.52(104)	178.95(89)	177.09(146)	176.17(82)	176.75(61)
C3-C4-C5-O5	-57.54(175)	-60.86(88)	-60.57(112)	-59.78(98)	-56.74(177)	-57.40(95)	-59.20(82)
O4-C4-C5-C6	62.03(205)	60.19(102)	58.04(129)	58.75(109)	53.73(189)	54.82(107)	58.14(89)
C3-C4-C5-C6	-173.40(246)	-178.73(88)	177.95(115)	-179.97(101)	179.9(164)	-178.75(83)	-177.81(64)
O5-C5-C6-O6	75.98(258)	73.76(106)	70.80(141)	71.81(118)	78.31(186)	83.07(97)	85.99(77)
C4-C5-C6-O6	-165.70(263)	-168.63(74)	-168.04(95)	-169.20(81)	-157.65(144)	-155.88(87)	-155.63(70)
C6-C5-O5-C1	-174.60(117)	-174.84(69)	-173.98(97)	-174.20(78)	-169.35(121)	-174.17(73)	-175.20(60)
C4-C5-O5-C1	61.77(119)	65.03(94)	62.65(119)	62.68(105)	68.26(169)	62.00(104)	63.36(89)
O1-C1-O5-C5	62.28(239)	63.47(81)	66.36(97)	64.53(90)	66.85(153)	65.24(81)	63.13(65)
C2-C1-O5-C5	-54.09(132)	-59.87(111)	-57.71(141)	-57.71(121)	-66.91(177)	-57.33(126)	-55.60(111)
O5-C1-O1-H8	32.27	29.94	28.82	29.91	54.75	58.02	59.86
C1-C2-O2-H9	-118.58	-113.08	-113.63	-113.94	78.44	70.61	69.51
C2-C3-O3-H10	-178.78	173.00	171.80	172.35	-126.73	-124.17	-123.33
C3-C4-O4-H11	171.18	172.86	174.23	172.49	7.93	8.00	9.25
C5-C6-O6-H12	178.72	-176.70	-174.97	-175.64	-111.67	-114.92	-116.37

Table S3. Geometry of OH···O hydrogen bonds at 0.0001-3.21 GPa range. All O-H bonds lengths have been normalized to the neutron-determined values according to Allan & Bruno.[7]

		0.0001	0.27(2)GPa	0.88(2)GPa	1.12(2)GPa	1.36(2)GPa	1.80(2)GPa	2.60(2)GPa	3.21(2)GPa
O1-H8···O5^{2564}	O···O	2.849	2.897(12)	2.811(5)	2.805(6)	2.798(6)	2.813(6)	2.718(19)	2.677(26)
	H···O	1.915	2.216	2.101	2.094	2.075	2.086	1.976	1.913
	C-O···O	96.2	93.3(7)	97.4(3)	96.1(4)	97.2(4)	96.0(4)	98.9(2)	100.5(15)
	O···O-C	169.6	154.1	159.8	158.1	158.5	156.5	155.7	156.5
	O-H···O	160.9	126.2	128.6	128.8	129.9	130.4	131.6	133.9
O1-H8···O5^{1554}	O···O	3.290	3.246(7)	3.172(5)	3.132(6)	3.119(7)	3.080(7)	3.000(7)	2.985(11)
	H···O	2.916	2.682	2.630	2.580	2.576	2.520	2.466	2.469
	C-O···O	157.2	156.2(1)	156.5(3)	155.9(4)	155.8(4)	154.7(5)	152.4(1)	150.0(14)
	O···O-C	79.6	110.5	105.8	107.1	107.0	107.5	110.0	109.5
	O-H···O	104.1	117.5	115.6	116.3	115.5	116.7	114.4	113.1
O2-H9···O3^{4557}	O···O	5.221	5.124(10)	4.997(6)	4.902(7)	4.892(8)	4.822(8)	4.621(19)	4.498(33)
	H···O	6.121	6.025	5.903	5.804	5.799	5.727	5.512	5.390
	C-O···O	80.8	81.7(5)	80.9(3)	82.3(4)	80.7(4)	81.1(4)	85.6(7)	84.1(10)
	O···O-C	139.6	139.8(4)	137.6(4)	138.6(4)	137.3(5)	138.0(5)	137.0(4)	135.0(8)
	O-H···O	19.7	19.9	19.1	19.7	19.0	19.2	21.3	20.6
O2-H9···O6^{3545}	O···O	2.778	2.810(12)	2.724(6)	2.749(7)	2.717(7)	2.688(8)	2.763(18)	2.768(30)
	H···O	1.821	1.855	1.766	1.790	1.765	1.730	1.794	1.801
	C-O···O	118.0	117.1(7)	116.7(3)	116.1(3)	117.6(4)	116.3(4)	110.6(12)	112.1(18)
	O···O-C	108.6	105.7(7)	106.3(3)	106.7(3)	106.70(4)	105.9(4)	99.0(9)	98.0(16)
	O-H···O	170.1	168.0	168.5	169.6	166.7	168.5	173.9	172.4
O3-H10···O1^{4456}	O···O	3.643	3.599(12)	3.493(6)	3.463(7)	3.400(7)	3.365(7)	3.228(16)	3.121(25)
	H···O	3.255	3.304	3.196	3.184	3.125	3.090	3.036	2.848
	C-O···O	163.8	162.7(5)	163.4(4)	162.9(4)	163.5(5)	162.6(5)	163.8(4)	161.7(10)
	O···O-C	71.1	70.8(5)	72.9(3)	73.5(3)	74.6(4)	74.8(4)	79.2(9)	81.3(13)
	O-H···O	105.9	99.9	99.8	98.4	98.1	98.0	92.3	96.9
O3-H10···O2^{4456}	O···O	2.708	2.700(14)	2.666(5)	2.646(5)	2.641(6)	2.620(6)	2.553(31)	2.481(52)
	H···O	1.758	1.822	1.773	1.765	1.753	1.732	1.681	1.596
	C-O···O	120.9	119.3(6)	117.2(3)	117.6(3)	117.8(4)	117.0(4)	118.4(11)	111.6(18)
	O···O-C	114.1	114.7(7)	112.8(3)	114.0(4)	112.8(4)	113.61(4)	118.5(11)	116.7(18)
	O-H···O	164.9	148.9	151.7	149.4	150.4	150.4	147.4	148.7
O4-H11···O2^{4456}	O···O	3.525	3.499(9)	3.364(6)	3.374(7)	3.361(7)	3.307(8)	3.257(17)	3.189(25)
	H···O	3.815	4.187	4.042	4.070	4.046	3.997	3.959	3.890
	C-O···O	106.4	105.8(5)	108.4(4)	107.8(4)	107.6(4)	108.0(4)	108.8(8)	109.0(12)
	O···O-C	152.5	153.8(6)	153.8(3)	155.9(4)	154.0(4)	154.9(5)	160.7(8)	160.0(13)
	O-H···O	65.5	39.9	40.5	39.2	39.9	39.6	38.5	38.7
O4-H11···O4^{2465}	O···O	2.777	2.781(6)	2.755(4)	2.734(5)	2.735(5)	2.722(5)	2.726(11)	2.746(15)
	H···O	1.819	2.100	2.055	2.030	2.028	2.008	1.985	1.999
	C-O···O	99.0	99.2(5)	97.3(4)	98.1(4)	96.9(4)	97.0(5)	98.9(9)	98.2(13)
	O···O-C	118.5	120.9(6)	118.6(4)	117.7(4)	119.5(4)	118.6(5)	118.2(17)	118.9(27)
	O-H···O	167.7	125.8	127.5	127.8	128.2	128.8	131.27	132.5
O6-H12···O3^{3556}	O···O	2.714	2.706(9)	2.679(6)	2.645(7)	2.652(7)	2.646(8)	2.597(11)	2.574(17)
	H···O	1.758	2.032	2.015	1.979	1.993	1.984	1.928	1.913
	C-O···O	101.1	100.3(4)	100.0(4)	101.3(4)	99.7(5)	99.9(5)	102.1(4)	103.5(7)
	O···O-C	129.4	131.0(7)	130.5(3)	129.6(3)	129.1(4)	129.1(4)	126.4(15)	131.3(25)
	O-H···O	169.7	124.9	123.9	123.8	123.3	123.4	124.0	123.2

O1···O3[4556]	O···O	3.643	3.599(12)	3.493(6)	3.463(7)	3.400(7)	3.365(7)	3.228(16)	3.121(25)
	C-O···O	71.1	70.8(5)	72.9(3)	73.5(3)	74.6(4)	74.8(4)	79.2(9)	81.3(13)
	O···O-C	163.8	162.7(5)	163.4(4)	162.9(4)	163.5(5)	162.6(5)	163.8(4)	161.7(10)
O1···O6[2564]	O···O	3.348	3.287(14)	3.235(5)	3.213(6)	3.188(6)	3.171(7)	2.986(23)	2.938(34)
	C-O···O	101.3	100.2(7)	104.7(3)	104.2(3)	106.5(4)	106.2(4)	112.6(15)	116.4(22)
	O···O-C	95.2	98.1(6)	97.4(3)	96.8(4)	96.9(4)	97.6(4)	103.0(7)	102.5(12)
O1···O6[2565]	O···O	5.116	5.007(10)	4.882(6)	4.831(7)	4.753(7)	4.702(8)	4.483(15)	4.382(22)
	C-O···O	33.0	31.0(6)	35.3(3)	34.8(3)	36.3(3)	36.1(3)	40.1(12)	42.7(18)
	O···O-C	126.0	129.1(4)	129.6(4)	128.5(4)	130.6(5)	131.4(5)	133.9(3)	132.9(5)
O6···O1[2564]	O···O	5.116	5.007(10)	4.882(6)	4.831(7)	4.753(7)	4.702(8)	4.483(15)	4.382(22)
	C-O···O	126.0	129.1(4)	129.6(4)	128.5(4)	130.6(5)	131.4(5)	133.9(3)	132.9(5)
	O···O-C	33.0	31.0(6)	35.3(3)	34.8(3)	36.3(3)	36.1(3)	40.1(12)	42.7(18)
O6···O1[2565]	O···O	3.348	3.287(14)	3.235(5)	3.213(6)	3.188(6)	3.171(7)	2.986(23)	2.938(34)
	C-O···O	95.2	98.1(6)	97.4(3)	96.8(4)	96.9(4)	97.6(4)	103.0(7)	102.5(12)
	O···O-C	101.3	100.2(7)	104.7(3)	104.2(3)	106.5(4)	106.2(4)	112.6(15)	116.4(22)

Contacts present in both, Phase I and II

Contacts present only in Phase I

Contacts present only in Phase II

O···O interactions

Table S3. (Continuation) Geometry of OH···O hydrogen bonds at 4.01-6.20 GPa range. All O-H bonds lengths have been normalized to the neutron-determined values according to Allan & Bruno.[7]

		4.00(2)GPa	4.20(2)GPa	5.15(2)GPa	5.23(2)GPa	5.33(2)GPa	5.54(2)GPa	5.80(2)GPa	6.20(2)GPa
O1-H8···O5[2564]	O···O	2.782(27)	2.688(26)	2.635(8)	2.636(7)	2.650(8)	2.637(13)	2.585(7)	2.585(6)
	H···O	2.049	1.933	1.867	1.863	1.881	1.674	1.621	1.619
	C-O···O	96.4(14)	96.9(13)	102.1(5)	101.0(4)	100.2(5)	108.6(8)	109.6(4)	109.2(3)
	O···O-C	150.8	153.3	152.4	150.8	151.8	160.2	161.4	160.9
	O-H···O	131.2	133.2	134.1	134.4	134.1	170.5	172.6	173.0
O1-H8···O5[1554]	O···O	2.933(12)	2.890(11)	2.834(12)	2.857(9)	2.853(11)	3.593(22)	3.475(10)	3.468(9)
	H···O	2.366	2.333	2.352	2.3516	2.329	3.374	3.272	3.258
	C-O···O	148.2(12)	148.5(12)	147.7(6)	147.4(5)	145.5(5)	119.3(9)	125.4(4)	126.1(3)
	O···O-C	109.8	106.9	108.8	109.8	109.3	104.7	101.9	102.2
	O-H···O	116.8	115.7	111.7	110.0	113.1	95.0	94.0	94.3
O2-H9···O3[4557]	O···O	4.311(37)	4.157(34)	4.235(11)	4.225(9)	4.217(9)	2.755(17)	2.735(8)	2.733(6)
	H···O	5.197	5.016	5.131	5.143	5.125	1.798	1.775	1.777
	C-O···O	86.3(10)	88.9(9)	80.7(6)	81.6(5)	81.4(6)	102.7(9)	102.8(4)	101.9(4)
	O···O-C	136.4(6)	134.5(5)	137.1(5)	137.0(4)	136.3(5)	137.9(8)	139.6(5)	139.1(4)
	O-H···O	22.1	24.6	18.8	18.6	18.5	169.2	169.7	168.2
O2-H9···O6[3545]	O···O	2.837(37)	2.919(37)	2.648(11)	2.652(9)	2.645(9)	3.306(17)	3.257(9)	3.223(8)
	H···O	1.872	1.974	1.696	1.690	1.687	4.099	4.057	4.018
	C-O···O	107.4(21)	101.4(19)	115.7(6)	116.1(5)	115.3(5)	109.7(9)	109.2(4)	109.8(4)
	O···O-C	104.2(21)	105.5(22)	104.4(7)	105.7(6)	105.0(6)	123.2(7)	123.9(4)	124.9(3)
	O-H···O	172.2	166.0	168.2	168.1	168.9	30.9	30.3	30.8

O3-H10···O1^{4456}	O···O	3.138(30)	3.050(28)	3.032(9)	3.021(8)	2.994(8)	2.625(13)	2.661(7)	2.657(6)
	H···O	2.876	2.864	2.816	2.819	2.803	1.724	1.779	1.774
	C-O···O	160.5(9)	163.0(6)	157.9(7)	158.5(6)	159.2(7)	126.9(12)	129.2(6)	129.2(5)
	O···O-C	82.7(13)	81.9(12)	84.7(6)	84.8(5)	86.4(5)	136.2(11)	128.3(5)	128.6(4)
	O-H···O	96.76	91.5	92.7	93.2	91.8	152.7	149.6	149.7
O3-H10···O2^{4456}	O···O	2.428(62)	2.235(59)	2.511(9)	2.531(7)	2.517(8)	3.203(2)	3.141(8)	3.104(7)
	H···O	1.563	1.378	1.656	1.633	1.640	2.608	2.539	2.501
	C-O···O	113.0(14)	114.6(12)	112.6(6)	112.7(5)	113.7(6)	87.2(9)	89.6(5)	89.7(4)
	O···O-C	121.0(19)	124.8(18)	115.8(4)	115.8(4)	115.8(4)	115.7(9)	115.6(5)	116.1(4)
	O-H···O	146.6	142.6	147.9	148.6	148.1	119.7	120.2	120.1
O4-H11···O2^{4456}	O···O	3.095(26)	3.017(24)	3.062(7)	3.072(6)	3.056(7)	2.744(14)	2.706(7)	2.691(6)
	H···O	3.821	3.754	3.771	3.758	3.760	1.797	1.753	1.737
	C-O···O	106.8(10)	105.1(10)	106.6(4)	107.0(4)	106.4(4)	119.4(7)	118.0(4)	117.9(4)
	O···O-C	162.7(9)	165.1(6)	155.3(7)	156.2(6)	156.4(6)	169.3(13)	169.6(6)	170.6(5)
	O-H···O	36.5	35.5	38.5	38.7	38.2	164.6	166.6	166.9
O4-H11···O4^{2465}	O···O	2.727(17)	2.722(17)	2.649(6)	2.650(5)	2.650(6)	3.379(15)	3.352(7)	3.330(6)
	H···O	1.962	1.955	1.898	1.894	1.893	4.212	4.185	4.166
	C-O···O	98.2(14)	97.6(15)	91.9(6)	91.9(5)	92.0(5)	80.4(9)	80.8(5)	79.5(4)
	O···O-C	119.7(21)	120.3(23)	121.6(7)	120.3(6)	120.9(6)	119.8(8)	120.5(5)	120.3(4)
	O-H···O	134.0	134.3	132.3	132.7	132.8	27.2	27.2	26.9
O6-H12···O3^{3556}	O···O	2.538(18)	2.560(21)	2.564(10)	2.574(8)	2.564(9)	2.753(14)	2.823(8)	2.813(8)
	H···O	1.868	1.910	1.929	1.917	1.922	1.792	1.866	1.861
	C-O···O	101.6(8)	102.9(10)	104.0(8)	103.6(6)	103.8(7)	104.1(11)	101.6(7)	100.5(6)
	O···O-C	128.7(24)	123.5(21)	126.5(7)	125.8(6)	124.5(6)	121.7(6)	119.0(4)	119.1(3)
	O-H···O	123.6	122.1	121.7	121.8	121.3	171.8	168.0	166.2
O1···O3^{4556}	O···O	3.138(30)	3.050(28)	3.032(9)	3.021(8)	2.994(8)	2.625(13)	2.661(7)	2.656(6)
	C-O···O	82.7(13)	81.9(12)	84.7(6)	84.8(5)	86.4(5)	136.2(11)	128.3(5)	128.6(4)
	O···O-C	160.5(9)	163.0(6)	157.9(7)	158.5(6)	159.2(7)	126.9(12)	129.2(6)	129.2(5)
O1···O6^{2564}	O···O	3.027(4)	2.982(43)	3.003(8)	3.002(7)	3.005(8)	3.598(19)	3.602(10)	3.617(8)
	C-O···O	114.0(20)	114.3(20)	120.1(5)	119.3(4)	120.4(4)	158.4(8)	155.2(4)	154.6(4)
	O···O-C	96.1(17)	93.2(18)	96.4(7)	96.4(6)	96.5(6)	80.3(9)	78.4(5)	76.7(4)
O1···O6^{2565}	O···O	4.305(26)	4.270(31)	4.081(12)	4.102(9)	4.074(10)	3.021(14)	3.043(8)	3.025(7)
	C-O···O	42.3(16)	42.3(17)	48.0(4)	47.2(4)	48.3(4)	82.7(10)	77.9(5)	77.6(4)
	O···O-C	132.0(9)	129.7(9)	132.0(6)	132.9(5)	132.6(5)	134.7(8)	134.8(5)	133.4(4)
O6···O1^{2564}	O···O	4.305(26)	4.270(31)	4.081(12)	4.102(9)	4.074(10)	3.021(14)	3.043(8)	3.025(7)
	C-O···O	132.0(9)	129.7(9)	132.0(6)	132.9(5)	132.6(5)	134.7(8)	134.8(5)	133.4(4)
	O···O-C	42.3(16)	42.3(17)	48.0(4)	47.2(4)	48.3(4)	82.7(10)	77.9(5)	77.6(4)
O6···O1^{2565}	O···O	3.027(4)	2.982(43)	3.003(8)	3.002(7)	3.005(8)	3.598(19)	3.602(10)	3.617(8)
	C-O···O	96.1(17)	93.2(18)	96.4(7)	96.4(6)	96.5(6)	80.3(9)	78.4(5)	76.7(4)
	O···O-C	114.0(20)	114.3(20)	120.1(5)	119.3(4)	120.4(4)	158.4(8)	155.2(4)	154.6(4)

Contacts present in both, Phase I and II

Contacts present only in Phase I

Contacts present only in Phase II

O···O interactions

Table S4. Geometry of CH···O hydrogen bonds at 0.0001-6.20 GPa range. All C-H bonds lengths have been normalized to the neutron-determined values according to Allan & Bruno.[7] Color legend has been included under Table S3.

		0.0001	5.33(2)GPa	5.54(2)GPa	6.20(2)GPa
C1-H1···O1^{1556}	C···O	3.596	2.902(14)	3.235(32)	2.954(14)
	H···O	3.503	2.590	2.549	2.270
	C-H···O	85.9	95.1	119.6	118.3
	C···O-C	171.6	171.1(5)	140.4(10)	147.6(4)
C1-H1···O 3^{4556}	C···O	3.453	3.211(10)	3.620(21)	3.713(10)
	H···O	2.854	2.787	3.803	3.921
	C-H···O	114.3	102.6	72.1	71.2
	C···O-C	141.5	134.4(6)	114.4(11)	113.2(5)
C1-H1···O5^{2565}	C···O	4.063	3.732(13)	3.458(21)	3.338(10)
	H···O	3.491	3.116	2.541	2.451
	C-H···O	114.0	116.1	140.5	136.7
	C···O-C	128.0	128.8	130.31	132.1
C1-H1···O6^{2565}	C···O	4.022	3.326(11)	3.115(13)	3.0531(6)
	H···O	3.074	2.237	2.244	2.239
	C-H···O	145.1	170.5	134.7	129.1
	C···O-C	122.8	128.0(5)	118.4(8)	113.7(4)
C2-H2···O1^{1556}	C···O	3.268	2.899(14)	3.02(24)	2.991(11)
	H···O	2.291	2.062	2.215	2.227
	C-H···O	147.6	130.7	128.0	124.6
	C···O-C	158.9	144.5(6)	127.5(8)	133.1(3)
C3-H3···O6^{3545}	C···O	3.528	3.284(11)	3.445(21)	3.433(10)
	H···O	2.927	2.700	2.639	2.656
	C-H···O	114.2	112.7	129.5	127.1
	C···O-C	151.0	148.6(5)	156.5(7)	156.3(4)
C4-H4···O4^{1556}	C···O	4.924	4.066(15)	3.736(30)	3.659(11)
	H···O	3.965	3.071	2.688	2.615
	C-H···O	146.6	151.1	159.2	158.5
	C···O-C	83.8	87.7(5)	104.1(10)	104.1(4)
C5-H5···O5^{1554}	C···O	4.935	4.017(14)	3.696(29)	3.597(12)
	H···O	3.975	3.012	2.653	2.555
	C-H···O	146.6	152.6	158.2	158.5
	C···O-C	85.2	88.0	86.4	86.7
C6-H6···O4^{2464}	C···O	3.983	3.418(13)	3.073(23)	3.121(9)
	H···O	3.412	2.698	2.255	2.379
	C-H···O	113.9	123.1	129.9	123.9
	C···O-C	119.3	114.4(6)	100.2(12)	99.0(5)
C6-H6···O4^{2465}	C···O	3.767	3.130(12)	2.856(22)	2.819(9)
	H···O	3.150	2.598	2.499	2.318
	C-H···O	116.5	109.2	97.7	105.8
	C···O-C	138.9	148.8(5)	167.4(12)	170.3(5)
C6-H7···O2^{3556}	C···O	3.223	2.872(12)	2.925(20)	2.890(9)
	H···O	2.732	2.328	2.354	2.382
	C-H···O	106.7	108.9	110.9	106.6
	C···O-C	102.1	105.1(6)	122.2(8)	120.9(4)
C6-H7···O6^{1556}	C···O	4.372	3.634(16)	3.526(30)	3.403(13)
	H···O	3.325	2.551	2.472	2.333
	C-H···O	160.2	171.8	162.0	166.2
	C···O-C	107.1	105.5(6)	112.0(10)	115.9(5)

Table S5. Geometry of H···H contacts at 0.0001-6.20 GPa range. All C-H and O-H bonds lengths have been normalized to the neutron-determined values according to Allan & Bruno.[7] Color legend has been included under Table S3.

	0.0001	5.33(2)GPa	5.54(2)GPa	6.20(2)GPa
H1···H8^{1556}	3.021	2.445	2.245	1.996
H1···H7^{2564}	2.373	2.662	3.702	3.683
H1···H12^{2565}	3.610	2.847	2.174	2.086
H2···H3^{1556}	3.371	2.493	1.917	1.895
H2···H5^{1556}	3.995	3.312	2.225	2.334
H2···H12^{3546}	2.693	1.975	2.666	2.554
H3···H4^{1554}	2.727	2.312	3.203	3.106
H3···H12^{3545}	2.691	2.034	2.248	2.193
H4···H5^{1556}	2.446	1.937	2.298	2.214
H4···H6^{2465}	3.651	2.862	1.909	1.966
H4···H11^{2465}	2.876	2.028	3.441	3.406
H5···H7^{1554}	2.676	2.379	3.211	3.102
H5···H11^{2464}	2.442	2.489	4.174	4.100
H6···H9^{3555}	2.589	2.401	4.454	4.451
H6···H11^{2465}	3.799	3.140	2.478	2.273
H7···H9^{3556}	2.712	2.142	2.744	2.816
H7···H11^{2465}	3.765	3.120	2.080	2.167
H8···H10^{4556}	3.641	3.668	2.138	2.217
H9···H10^{4557}	5.879	4.700	2.205	2.220
H9···H10^{4556}	2.364	2.120	2.761	2.648
H9···H11^{4556}	3.782	3.722	1.886	1.813
H9···H12^{3545}	2.327	1.578	4.530	4.421
H10···H12^{3546}	2.365	2.691	2.105	2.201
H11···H11^{2464}	2.500	2.140	4.854	4.807

Table S6. ORTEP symmetry code.[2]

ORTEP code	Symmetry code	ORTEP code	Symmetry code
1554	x, y, -1+z	3545	-x, -½+y, ½-z
1556	x, y, 1+z	3546	-x, -½+y, 1½-z
2464	-½-x, 1-y, -½+z	3556	-x, ½+y, 1½-z
2465	-½-x, 1-y, ½+z	4456	-½+x, ½-y, 1-z
2564	½-x, 1-y, -½+z	4557	½+x, ½-y, 2-z
2565	½-x, 1-y, ½+z	4556	½+x, ½-y, 1-z

Table S7. Compressibility parameters β of unit-cell parameters a, b, c and volume V, calculated as $\beta_x = -\frac{1}{x}\frac{\partial x}{\partial p}$, where x is a given parameter. The differential part was analytically calculated based on a quadratic polynomial fitted to the experimentally obtained pressure dependences of unit-cell volume V and parameters a, b, c for α-D-glucose Phase I and linear function for Phase II. Used functions are listed in Table S8. Compressibility parameters β at 0.1 MPa were calculated based on the structural information obtained by Brown and Levy.[1]

Pressure [GPa]	β_a[GPa^{-1}]	β_b[GPa^{-1}]	β_c[GPa^{-1}]	β_V[GPa^{-1}]
0.0001	$13.92*10^{-3}$	$1.37*10^{-3}$	$30.89*10^{-3}$	$45.33*10^{-3}$
0.27	$13.13*10^{-3}$	$1.32*10^{-3}$	$31.21*10^{-3}$	$44.55*10^{-3}$
0.88	$11.32*10^{-3}$	$1.21*10^{-3}$	$31.91*10^{-3}$	$42.71*10^{-3}$
1.12	$10.61*10^{-3}$	$1.16*10^{-3}$	$32.23*10^{-3}$	$42.05*10^{-3}$
1.36	$9.86*10^{-3}$	$1.12*10^{-3}$	$32.40*10^{-3}$	$41.01*10^{-3}$
1.80	$8.52*10^{-3}$	$1.04*10^{-3}$	$32.75*10^{-3}$	$39.25*10^{-3}$
2.60	$6.04*10^{-3}$	$0.89*10^{-3}$	$33.56*10^{-3}$	$36.00*10^{-3}$
3.21	$4.13*10^{-3}$	$0.77*10^{-3}$	$33.88*10^{-3}$	$33.06*10^{-3}$
4.01	$1.59*10^{-3}$	$0.62*10^{-3}$	$34.77*10^{-3}$	$29.25*10^{-3}$
4.20	$0.99*10^{-3}$	$0.58*10^{-3}$	$35.03*10^{-3}$	$28.40*10^{-3}$
5.15	$-2.04*10^{-3}$	$0.41*10^{-3}$	$35.95*10^{-3}$	$23.36*10^{-3}$
5.23	$-2.29*10^{-3}$	$0.39*10^{-3}$	$36.07*10^{-3}$	$22.95*10^{-3}$
5.33	$-2.61*10^{-3}$	$0.37*10^{-3}$	$36.11*10^{-3}$	$22.34*10^{-3}$
5.54	$3.68*10^{-3}$	$6.41*10^{-3}$	$13.06*10^{-3}$	$23.74*10^{-3}$
5.80	$3.68*10^{-3}$	$6.42*10^{-3}$	$13.14*10^{-3}$	$23.88*10^{-3}$
6.20	$3.69*10^{-3}$	$6.44*10^{-3}$	$13.18*10^{-3}$	$24.12*10^{-3}$

Table S8. Quadratic polynomial (α-D-glucose Phase I) and linear (α-D-glucose Phase II) functions fitted to the experimentally obtained pressure dependences of unit-cell volume V and parameters a, b, c of α-D-glucose used for the calculation of the differential part of compressibility parameter $\beta_x = -\frac{1}{x}\frac{\partial x}{\partial p}$.

	Phase I	Phase II
$a(p)$	$a = 0.016\,p^2 - 0.1443\,p + 10.373$ (R= 0.9942)	$a = -0.03576\,p + 9.921843$ (R= 0.9942)
$b(p)$	$b = 0.0014\,p^2 - 0.0204\,p + 14.852$ (R= 0.9321)	$b = -0.09616\,p + 15.53217$ (R= 0.9321)
$c(p)$	$c = 0.0042\,p^2 - 0.1537\,p + 4.9578$ (R= 0.9971)	$c = -0.05565\,p + 4.565107$ (R= 0.9971)
$V(p)$	$V = 1.9295\,p^2 - 34.717\,p + 763.59$ (R= 0.9981)	$V = -14.7437\,p + 702.768$ (R= 0.9981)

References:

1. G. M. Brown and H. A. Levy, *Acta Crystallogr., Sect. B: Struct. Sci.*, 1979, **35**, 656.
2. C. K. Johnson, ORTEPII. Report ORNL-5138, TN: Oak Ridge National Laboratory, Memphis, 1976.
3. A. Bondi, J. Phys. Chem., 1964, **68**, 441.
4. M. A. Spackman, J. J. McKinnon, *CrystEngComm*, 2002, **4**, 378.
5. M. A. Spackman, D. Jayatilaka, *CrystEngComm*, 2009, **11**,19.
6. C. F. Macrae, I. J. Bruno, J. A. Chisholm, P. R. Edgington, P. McCabe, E. Pidcock, L. Rodriguez-Monge, R. Taylor, J. van de Streek, P. A. Wood, *J. Appl. Crystallogr.*, 2008, **41**, 466.
7. F. H. Allen, I. J. Bruno, *Acta Crystallogr., Sect. B: Struct. Sci*., 2010, **66**, 380.
